# Participation of MicroRNAs in the Treatment of Cancer with Phytochemicals

**DOI:** 10.3390/molecules25204701

**Published:** 2020-10-14

**Authors:** Seung Wan Son, Han Yeoung Lee, Sokviseth Moeng, Hyo Jeong Kuh, Soo Young Choi, Jong Kook Park

**Affiliations:** 1 Department of Biomedical Science and Research Institute for Bioscience & Biotechnology, Hallym University, Chunchon 24252, Korea; miyanae@naver.com (S.W.S.); gksdudsd@gmail.com (H.Y.L.); sokvisethmoeng@yahoo.com (S.M.); sychoi@hallym.ac.kr (S.Y.C.); 2 Department of Medical Life Sciences, College of Medicine, The Catholic University of Korea, Seoul 06591, Korea; hkuh@catholic.ac.kr

**Keywords:** non-coding RNA, microRNA, cancer, phytochemical, natural compound

## Abstract

Cancer is a global health concern and one of the main causes of disease-related death. Even with considerable progress in investigations on cancer therapy, effective anti-cancer agents and regimens have thus far been insufficient. There has been compelling evidence that natural phytochemicals and their derivatives have potent anti-cancer activities. Plant-based anti-cancer agents, such as etoposide, irinotecan, paclitaxel, and vincristine, are currently being applied in medical treatments for patients with cancer. Further, the efficacy of plenty of phytochemicals has been evaluated to discover a promising candidate for cancer therapy. For developing more effective cancer therapy, it is required to apprehend the molecular mechanism deployed by natural compounds. MicroRNAs (miRNAs) have been realized to play a pivotal role in regulating cellular signaling pathways, affecting the efficacy of therapeutic agents in cancer. This review presents a feature of phytochemicals with anti-cancer activity, focusing mainly on the relationship between phytochemicals and miRNAs, with insights into the role of miRNAs as the mediators and the regulators of anti-cancer effects of phytochemicals.

## 1. Introduction

Cancer is generally incurable and life-threatening. According to the GLOBOCAN database, there were approximately 18.1 million new cancer cases and 9.6 million cancer deaths worldwide in 2018 [1]. Chemotherapy and radiotherapy are currently used in cancer treatments; however, they are moderately effective, leading to the insufficient regression and remission of cancer. Moreover, the adverse effects of chemotherapy, such as toxicity to normal cells and therapeutic resistance, restrict the utilization of anti-cancer agents [2,3,4]. Therefore, intensive efforts have been made to continuously search for and develop new agents and strategies for cancer therapy with better efficacy.

Natural compounds, procured from several resources, have significant therapeutic potentials for diverse types of diseases, including cancer, and provide critical guides for drug discovery. For example, psammaplin A and didemnin B from marine organisms show anti-cancer properties and effectively induce apoptosis [5]. Aphidicolin is a metabolite from fungi, such as *Harziella entomophilla*, and exhibits anti-cancer activity by inhibiting DNA replication and sensitizing cancer cells to therapeutic agents, such as fludarabine and cladribine in chronic lymphocytic leukemia [6]. In addition, various plant-derived phytochemicals and their derivatives possess a high potential as anti-cancer agents and are still under evaluation. Mounting evidence from preclinical studies demonstrated that phytochemical compounds effectively repress cancer progression and malignancy, such as cell proliferation, growth, invasion, and metastasis [7,8]. Additionally, anti-cancer agents currently used in clinical therapy are derived from plants. Well-known examples include etoposide, irinotecan, paclitaxel, and vincristine. Primary molecular targets of irinotecan and etoposide are topoisomerase I and topoisomerase II, respectively [9,10]. Paclitaxel and vincristine are microtubule inhibitors, displaying anti-cancer properties, such as cell cycle arrest and apoptosis [10,11].

MicroRNAs (miRNAs) are small regulatory non-coding RNAs that can affect multiple cellular signaling pathways by controlling the degradation and translation of their target messenger RNAs (mRNAs). Since miRNA levels can be epigenetically, transcriptionally, and/or post-transcriptionally regulated, miRNAs are able to mediate the effects of several stimuli such as a drug. For example, paclitaxel elevates the level of miR-512-3p that targets an anti-apoptotic gene, FADD-like apoptosis regulator (CFLAR, also known as c-FLIP), suggesting that miR-512-3p is responsible for paclitaxel-induced apoptosis to a degree in hepatocellular carcinoma cells [12]. In terms of cancer, miRNAs serve as oncogenic or tumor-suppressive factors depending on their target genes and cell types [4,13,14]. Additionally, dysregulated miRNAs can influence the sensitivity of cancer cells to anti-cancer treatments by modulating cellular events, such as apoptosis, autophagy, stemness, drug efflux, drug metabolism, and epithelial-mesenchymal transition (EMT) [4,15].

An improved understanding of the mode of action of phytochemicals at the molecular levels will be beneficial to establish novel and effective therapeutic strategies to strive against cancer. This article aims to present a review of the role of miRNAs in mediating and regulating the anti-cancer effects of phytochemicals. In the case of plant-derived compounds currently applied in clinical therapy, we focused on the involvement of miRNAs in the occurrence of resistance to these compounds. The phytochemical compounds and their derivatives mentioned in this review are summarized in Table 1 and Table 2.

## 2. Oncogenic MiRNAs Inhibited by Phytochemicals Currently Evaluated in Preclinical Studies and Clinical Trials

### 2.1. MiRNAs and Nitrogen-Containing Compounds

#### 2.1.1. Berberine and Evodiamine

**Figure 1:**
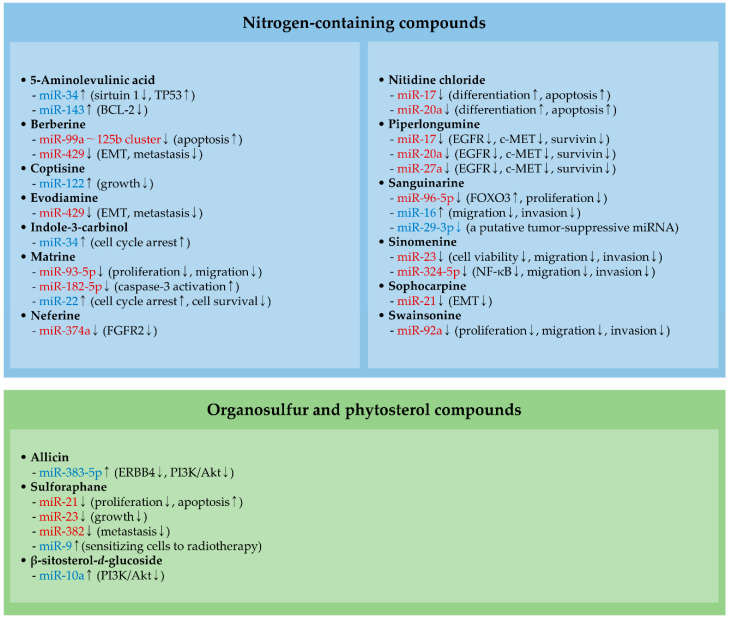
Effects of nitrogen-containing compounds and organosulfur/phytosterol compounds on the expression level of oncogenic miRNAs (red) and tumor-suppressive miRNAs (blue). Arrows indicate the upregulation (↑) and downregulation (↓) of miRNA levels and consequential effects on cancer. The role of miRNAs in cancer therapy with phytochemicals is described in Section 2 and Section 3.

The miR-99a–125b cluster located at chromosome 21 consists of three miRNAs, namely miR-99a, let-7c, and miR-125b. These miRNAs have been validated as oncogenic or tumor-suppressive miRNAs depending on the type of cancer. For example, miR-99a can inhibit proliferation, migration, and invasion by directly regulating fibroblast growth factor receptor 3 (FGFR3) in breast cancer [87]. In multiple myeloma (MM), miR-125b is known to suppress apoptosis induced by dexamethasone via targeting tumor protein p53 (TP53) [88]. Recently, it was demonstrated that the levels of miR-99a–125b are downregulated by berberine treatments and that the knockdown of miR-99a–125b causes cell cycle arrest as well as apoptosis induction in MM [17] (Figure 1 and Table 3).

One of the critical miRNAs involved in the progression of colorectal cancer is miR-429. It has been noticed that this miRNA is overexpressed in colorectal cancer tissues compared to their normal counterparts and that miR-429 is able to augment EMT and metastasis of colorectal cancer by modulating the expression of homeobox A5 (HOXA5) [89]. Additionally, treatments of colorectal cancer with either berberine or evodiamine result in a decrease in miR-429 expression [90], suggesting that the anti-cancer activity of both phytochemicals is partly mediated by the modulation of miR-429 levels (Figure 1 and Table 3).

#### 2.1.2. Matrine

Matrine has been reported to elicit anti-cancer effects on multiple cancer types. For example, matrine suppresses the migration and invasion capacities of lung, prostate, and breast cancer cells via downregulating the levels of C-X-C motif chemokine receptor 4 (CXCR4) [107]. In addition, it was demonstrated that matrine represses miR-182-5p and miR-93-5p levels in papillary thyroid and gastric cancer cells, respectively, and retards cancer growth in vivo [21,96] (Figure 1 and Table 3). In particular, the overexpression of miR-182-5p (a member of the miR-183-96-182 cluster) can block the matrine-mediated activation of caspase-3 in papillary thyroid cancer cells [21]. In addition, miR-93-5p (a member of the miR-106b-25 cluster) positively affects cell proliferation and the migration of gastric cancer cells via directly targeting AHNAK nucleoprotein (AHNAK), which is a negative regulator of the EMT process [96,108].

#### 2.1.3. Neferine

Fibroblast growth factor receptor 2 (FGFR2) is known to be overexpressed in subsets of breast cancer tissues and is negatively correlated with the overall survival of breast cancer patients [109,110]. It was recently shown that treatments with neferine restrain the proliferation, migration, and invasion of breast cancer cells via downregulating miR-374a, which positively controls FGFR2 levels [22] (Figure 1 and Table 3). Neferine has been shown to sensitize cancer cells to oxaliplatin and tumor necrosis factor-related apoptosis-inducing ligand (TRAIL) in hepatocellular carcinoma and prostate cancer, respectively [111,112]. Since FGFR2 can confer tamoxifen resistance in estrogen-positive breast cancer cells [113], it is feasible that neferine also reverses FGFR2-mediated tamoxifen resistance.

#### 2.1.4. Nitidine Chloride and Swainsonine

c-Myc, an oncogenic transcription factor, positively controls cell proliferation and decelerates the senescence and differentiation of leukemia [114,115,116]. The miR-17–92 cluster containing six different miRNAs (miR-17, miR-18a, miR-19a, miR-19b-1, miR-20a, and miR-92a) is transcriptionally induced by c-Myc, contributing to the maintenance of cell proliferation and survival via targeting multiple genes, including BCL2-like 11 (BCL2L11, also known as Bim) in leukemia [117]. A recent study demonstrated that nitidine chloride attenuates c-Myc expression levels via ubiquitin-mediated degradation and induces differentiation and apoptosis by upregulating cyclin-dependent kinase inhibitor 1A (CDKN1A, also known as p21Cip1) in leukemia. Indeed, nitidine chloride reduces miR-17 and miR-20a levels. The overexpression of these miRNAs reduces CDKN1A levels in leukemia cells, indicating that the anti-cancer activity of nitidine chloride is mediated by the c-Myc-miR-17–92 axis [91] (Figure 1 and Table 3).

In glioma, miR-92a has been identified to accelerate proliferation, cell survival, and metastasis through regulating BCL2L11, cadherin 1 (CDH1, also known as E-cadherin) and the Akt/mechanistic target of rapamycin kinase (mTOR) signaling [118,119,120]. It was shown that the treatment of glioma cells with swainsonine hinders cell proliferation, migration, and invasion via downregulating miR-92a levels and Akt/mTOR activities [28] (Figure 1 and Table 3).

#### 2.1.5. Piperlongumine

Piperlongumine has been identified to induce reactive oxygen species (ROS), thus exhibiting intense anti-cancer activity and sensitizing cells to anti-cancer agents such as paclitaxel [24,121]. Increased ROS following a piperlongumine treatment diminishes the expression levels of both c-Myc and c-Myc-regulated miRNAs (miR-17, miR-20a, and miR-27a) [24] (Figure 1 and Table 3). Downregulation of these miRNAs further leads to the induction of their target mRNAs, zinc finger and BTB domain-containing protein 4 (ZBTB4) and ZBTB10. Both ZBTB4 and ZBTB10 are negative regulators of specificity protein (SP) transcription factors, such as SP1 and SP3. Thus, piperlongumine can inhibit several oncogenic factors, such as the epidermal growth factor receptor (EGFR), c-MET, and survivin, which are transcribed by SP transcription factors [24].

#### 2.1.6. Sanguinarine

Sanguinarine has been shown to have anti-cancer activities against several types of cancer. For instance, sanguinarine can suppress proliferation and cell viability by regulating the levels of ROS and pro-apoptotic genes in cervical cancer cells [122]. Sanguinarine also impedes EMT by inactivating the Wingless (Wnt)/β-catenin and transforming growth factor-beta (TGF-β) signaling pathways in colorectal cancer and hepatocellular carcinoma, respectively [123,124]. In gastric cancer, sanguinarine blocks cell proliferation, together with a decrease in miR-29-3p and miR-96-5p levels [25] (Figure 1, Table 3 and Table 4). MiR-96-5p has been found to accelerate proliferation via negatively modulating forkhead box O3 (FOXO3), which can induce apoptotic cell death [125,126]. In the case of miR-29-3p, overexpression of miR-29-3p remarkably restrains the migration and invasion of gastric cancer cells by suppressing multiple oncogenes such as FGFR4 [127,128], implying that sanguinarine can unexpectedly downregulate tumor-suppressive miRNAs.

#### 2.1.7. Sinomenine

In prostate cancer, sinomenine inactivates the phosphoinositide 3-kinase (PI3K)/Akt and Janus kinase (JAK)/signal transducer and activator of transcription 3 (STAT3) signal pathways, leading to the inhibition of cell viability, migration, and invasion [26]. In that study, it was noted that miR-23 is downregulated in sinomenine-treated cells and that the ectopic introduction of miR-23 reverses the anti-cancer effects of sinomenine on prostate cancer cells [26]. Another study also showed that the activity of nuclear factor kappa B (NF-κB) is dampened by sinomenine, thus reducing invasion and migration of breast cancer cells [104]. Furthermore, it is noteworthy that miR-324-5p levels are decreased by sinomenine treatments and that this miRNA directly targets the CUE domain-containing 2 (CUEDC2), a negative regulator of NF-κB [104] (Figure 1 and Table 3).

#### 2.1.8. Sophocarpine

One of the most enormously explored oncogenic miRNAs is miR-21. The levels of miR-21 are upregulated in several cancers and are correlated with metastasis status [155,156]. In addition, miR-21 promotes EMT via negatively regulating several EMT-inhibiting factors, including phosphatase and tensin homolog (PTEN), SRY-box transcription factor 17 (SOX17), and leucine zipper transcription factor-like 1 (LZTFL1) [155,157,158]. Interestingly, cell-free assays indicated that sophocarpine binds to miR-21 precursors and blocks the Dicer-mediated maturation of miR-21; therefore, sophocarpine can inhibit the progression of head and neck cancer by negatively modulating EMT [27] (Figure 1 and Table 3).

### 2.2. MiRNAs and an Organosulfur Compound

#### Sulforaphane

Several studies demonstrated that miRNAs involve the regulation of cell cycle, senescence, and apoptosis in sulforaphane-treated cancer cells [30,93,94]. In glioblastoma, sulforaphane suppresses the activity of transcription factor 4 (TCF4), a downstream factor of Wnt/β-catenin signaling, thereby reducing the levels of miR-21 [93]. Such downregulation of miR-21 can potentiate temozolomide-induced apoptosis when combined with sulforaphane, suggesting that sulforaphane can be considered as a promising adjuvant candidate for glioblastoma therapy [93] (Figure 1 and Table 3).

Sulforaphane also shows anti-cancer efficacy by suppressing cell proliferation and inducing apoptosis in colorectal cancer. In that study, it was demonstrated that sulforaphane reduces the expression of both miR-21 and telomerase reverse transcriptase (TERT) [30]. TERT is generally recognized to exhibit multiple oncogenic activities, contributing to the regulation of angiogenesis, stemness, EMT, and metastasis [159]. Downregulation of TERT levels can be due to the ability of sulforaphane to turn off the transcription of TERT by recruiting methyl-CpG binding protein 2 (MECP2) to the TERT promoter [160]. Another possibility is that TERT levels can be positively controlled by miR-21 since TERT transcription is mediated by ERK1/2 signaling activated by this miRNA [161,162] (Figure 1 and Table 3).

In breast cancer, sulforaphane was noticed to promote cell cycle arrest, cellular senescence, and apoptosis induction by modulating global DNA methylation status and miRNA levels. For example, oncogenic miRNAs (miR-23 and miR-382) are downregulated by the treatment with sulforaphane [94]. It has been demystified that knockdown of miR-23 abates the growth of breast cancer in vivo and that miR-382 triggers breast cancer metastasis by activating Ras/ERK signaling pathways via targeting RAS-like estrogen-regulated growth inhibitor (RERG) [163,164] (Figure 1 and Table 3).

### 2.3. MiRNAs and Phenolic Compounds

#### 2.3.1. Baicalin

**Figure 2:**
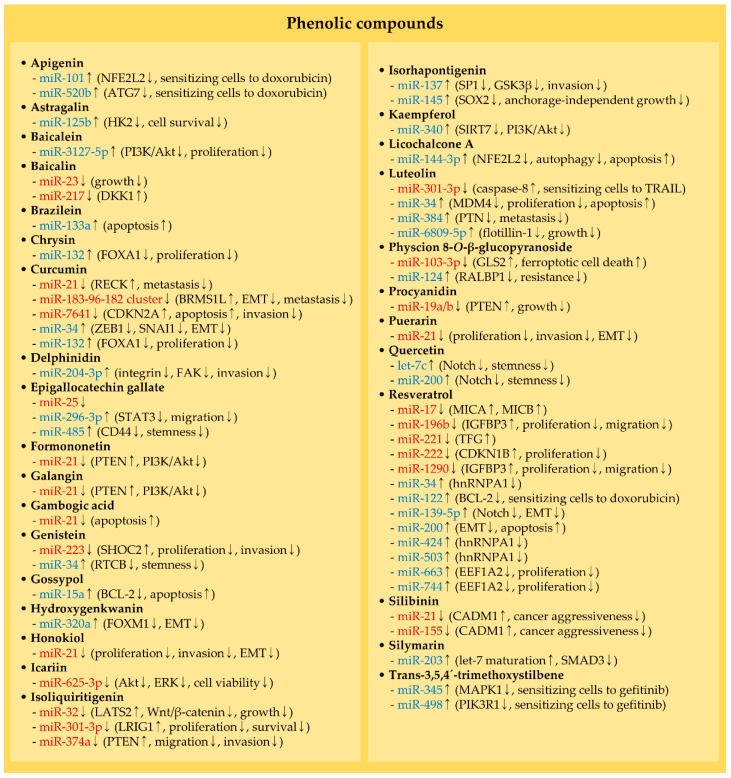
Effects of phenolic compounds on the expression level of oncogenic miRNAs (red) and tumor-suppressive miRNAs (blue). Arrows indicate the upregulation (↑) and downregulation (↓) of miRNA levels and consequential effects on cancer. The role of miRNAs in cancer therapy with phytochemicals is described in Section 2 and Section 3.

Apoptotic cell death is induced by baicalin treatments, and further evidence showed that various oncogenic miRNAs, such as miR-23 and miR-217, can be depleted by baicalin in colorectal cancer [36,99] (Figure 2 and Table 3). Baicalin impedes the growth of colorectal cancer in vivo via repressing c-Myc expression in conjunction with a reduction of miR-23, which is one of the c-Myc-regulated miRNAs [36]. Additionally, baicalin can induce apoptosis through subduing Wnt/β-catenin signaling, accompanied by the upregulated levels of Dickkopf-related protein 1 (DKK1), an endogenous inhibitor of Wnt signaling [99]. In this study, it was noted that baicalin downregulates the expression of miR-217, which undeviatingly targets DKK1.

#### 2.3.2. Curcumin

Reversion-inducing cysteine-rich protein with Kazal Motifs (RECK) is known to suppress proliferation, invasion, and metastasis primarily through inhibiting matrix metalloproteinases (MMPs), such as MMP-2 and MMP-9, in multiple cancer types [165,166,167]. A recent study demonstrated that the treatment of osteosarcoma cells with curcumin decelerates cell proliferation, along with a decrease in miR-21, which targets RECK [39]. The miR-21/RECK axis can support the previous finding that curcumin inhibits cancer metastasis [168,169] (Figure 2 and Table 3).

It is acknowledged that human growth hormone (hGH) facilitates proliferation, survival, EMT, etcetera, in cancer [170,171,172]. In breast cancer, hGH is capable of increasing the levels of the miR-183-96-182 cluster members via transcription factors, such as signal transducer and activator of transcription 3 (STAT3) and STAT5 [170]. This miR-183-96-182 cluster is overexpressed in breast cancer and prompts proliferation and migration [173]. Additionally, these cluster members facilitate cancer invasion, EMT, and metastasis through commonly targeting breast cancer metastasis-suppressor 1-like (BRMS1L) [170]. Recent evidence suggested that curcumin can block the expression of miR-183-96-182 cluster induced by hGH, ultimately preventing hGH-mediated breast cancer aggressiveness [97] (Figure 2 and Table 3).

Besides, curcumin diminishes the expression of miR-7641, which directly controls CDKN2A (also called p16Ink4A) [106]. This study showed that curcumin weakens bladder cancer invasion coupled with apoptosis induction by elevating CDKN2A levels [106]. CDKN2A has been recognized to negatively regulate metastasis-related signaling and induce apoptosis following cell cycle arrest [174,175] (Figure 2 and Table 3).

#### 2.3.3. Epigallocatechin Gallate

Members of the miR-106b–25 cluster (miR-25, miR-93, and miR-106b) have been realized to contribute to the progression of breast cancer. For example, miR-106 potentiates the metastatic potential of breast cancer by increasing the activity of Rho-associated coiled-coil containing protein kinase 1 (ROCK1) [176]. Moreover, miR-25 can inhibit apoptotic cell death by suppressing B-cell translocation gene 2 (BTG2), a negative regulator of Akt and ERK [177]. Recently, it was found that miR-25 is suppressed by epigallocatechin gallate (EGCG) in breast cancer cells, contributing to the induction of apoptosis in vitro and the retardation of cancer growth in vivo [95] (Figure 2 and Table 3).

#### 2.3.4. Formononetin, Galangin, Gambogic Acid, Honokiol, and Puerarin

By suppressing miR-21, diverse phytochemicals can exert anti-cancer effects. As stated in Section 2.1.8, miR-21 directly modulates PTEN, whose function is to inactivate PI3K/Akt signaling in cancer. As a consequence of the miR-21 inhibition, formononetin and galangin upregulate PTEN levels in bladder and cholangiocarcinoma, respectively, thereby inhibiting cancer aggressiveness [42,43]. In colorectal cancer, gambogic acid induces apoptosis by suppressing miR-21 expression, and the cytotoxicity of gambogic acid is reversed by overexpressing miR-21 [44]. Additionally, honokiol and puerarin act as miR-21 repressors and suppress multiple cellular events, such as proliferation, invasion, and EMT [48,57] (Figure 2 and Table 3).

#### 2.3.5. Genistein

F-Box and WD repeat domain containing 7 (FBXW7), a tumor-suppressive E3 ligase, is known to degrade SHOC2 leucine-rich repeat scaffold protein (SHOC2), which activates Ras/ERK signaling in pancreatic cancer [178]. Recently, it was investigated that exposure to genistein attenuates pancreatic cancer proliferation and invasion by downregulating miR-223, which targets SHOC2 [101] (Figure 2 and Table 3). The transcription of miR-223 is enhanced by CCAAT/enhancer-binding protein beta (CEBPB), and genistein can inhibit CEBPB [179,180]. Therefore, it is feasible that genistein can reduce miR-223 levels via a CEBPB downregulation.

#### 2.3.6. Icariin, Isoliquiritigenin, and Luteolin

The anti-cancer effects of icariin were assessed in thyroid cancer cells [49]. It showed that cell viability is suppressed in icariin-treated cells, concomitantly with a decrease in migration and invasion of cancer cells. After treatment with icariin, miR-625-3p levels are decreased, along with the inactivation of Akt and ERK (Figure 2 and Table 3). Ectopic introduction of miR-625-3p reactivates both Akt and ERK, indicating that miR-625-3p serves as an oncogenic factor [49]. However, miR-625-3p can negatively affect the proliferation and metastasis of gastric cancer cells [181], implying that the function of miR-625-3p is cell-type dependent.

Recent reports have demonstrated that anti-cancer activity of isoliquiritigenin is accomplished by downregulating oncogenic miRNAs. The expression of miR-32 is reduced by isoliquiritigenin in nasopharyngeal cancer cells, hence increasing the levels of large tumor suppressor kinase 2 (LATS2), a target mRNA of miR-32. LATS2 is responsible for the reduction of cancer growth by inhibiting Wnt/β-catenin signaling [50]. Further, it was observed that the proliferation and survival of melanoma cells are retarded by isoliquiritigenin and that one of the significantly downregulated miRNAs is miR-301-3p. The evaluation of miR-301-3p activity indicated that this miRNA functions as an oncogene by targeting leucine-rich repeats and immunoglobulin-like domains protein 1 (LRIG1) [102]. Furthermore, PTEN is upregulated by isoliquiritigenin, owing to the reduction of miR-374a levels, hence subduing the migration and invasion of breast cancer cells [105] (Figure 2 and Table 3).

Luteolin causes cell cycle arrest, induces apoptosis, and inhibits metastasis via repressing the expression of oncogenic factors, such as TERT, c-Myc, and MMPs [150,182]. Besides, luteolin can sensitize ovarian cancer and breast cancer cells to cisplatin and tamoxifen, respectively [183,184]. Moreover, luteolin diminishes the levels of miR-301-3p that targets caspase-8, ultimately sensitizing pancreatic cancer cells to the TNF superfamily member 10 (TNFSF10, also known as TNF-related apoptosis-inducing ligand (TRAIL)) [103] (Figure 2 and Table 3).

#### 2.3.7. Physcion 8-*O*-β-Glucopyranoside and Procyanidin

Ferroptotic cell death can be induced by iron-dependent lipid peroxidation. Additionally, it was denoted that glutaminolysis catalyzed by glutaminase 2 (GLS2) facilitates ferroptotic cell death [185,186]. Interestingly, physcion 8-*O*-β-glucopyranoside (PG) is able to cause the ferroptotic cell death of gastric cancer cells by downregulating the levels of miR-103-3p and upregulating GLS2, a target of miR-103-3p [55] (Figure 2 and Table 3). Unlike wild-type TP53, mutant TP53 is incapable of regulating GLS2 transcription [89]. Since TP53 is widely mutated in cancer, it can be postulated that the miR-103-3p-GLS2 axis is an alternative way to modulate the ferroptotic cell death of cancer cells.

In lung cancer, miR-19a and miR-19b, members of the miR-17–92 cluster, were evaluated to be lowered by procyanidin. Also, procyanidin causes growth retardation of lung cancer in vivo, along with an increase in the level of PTEN, owing to the reduction of these miRNAs [56] (Figure 2 and Table 3). Indeed, miR-19a and miR-19b are validated miRNAs that target PTEN [187,188].

#### 2.3.8. Resveratrol

Resveratrol has been shown to exhibit multitudinous anti-cancer effects owing to its ability to inhibit diverse signaling pathways involved in cell growth, apoptosis, and migration. For example, resveratrol has been known to inhibit c-Myc, thereby inducing cell cycle arrest and apoptotic cell death [189,190]. In breast cancer, resveratrol was found to promote the lysis of cancer cells by natural killer (NK) cells in vitro and in vivo via suppressing the expression of miR-17 in a c-Myc-dependent manner. In that study, it was unveiled that miR-17 directly targets MHC class I polypeptide-related sequence A (MICA) and MICB, which are ligands for NK cells [92]. In acute lymphoblastic leukemia, resveratrol inhibits cell proliferation and viability by downregulating the levels of miR-196b and miR-1290, both of which directly regulate the expression of insulin-like growth factor-binding protein 3 (IGFBP3) [98] (Figure 2 and Table 3).

Resveratrol obstructs the activation of NF-κB, thereby inhibiting the levels of anti-apoptotic and pro-proliferative genes, such as B-cell CLL/Lymphoma 2 (BCL-2) and cyclin D1 [191,192]. Moreover, in melanoma, the anti-cancer activity of resveratrol is mediated by downregulating the NF-κB activity and the levels of NF-κB-regulated miR-221, which targets the tropomyosin-receptor kinase fused gene (TFG) [59]. While TFG was supposed to be a tumor-suppressive gene in melanoma [193], its role in melanoma has been understudied. Therefore, it is still further required to investigate the function of TFG. In thyroid cancer, it was demonstrated that miR-222 is downregulated by resveratrol in a cell-line dependent manner [100]. The seed sequence of miR-222 is identical to miR-221; therefore, miR-221 and miR-222 can target the same mRNAs. For example, miR-221 and miR-222 can facilitate cell proliferation by targeting CDKN1B (also known as p27Kip1) in thyroid cancer cells [194] (Figure 2 and Table 3).

#### 2.3.9. Silibinin

Both miR-21 and miR-155 can promote cancer aggressiveness via commonly targeting a tumor-suppressive gene such as cell adhesion molecule 1 (CADM1) [195,196]. Additionally, these two miRNAs were identified as possible biomarkers for breast cancer since their levels in plasma from cancer patients display the correlation with the degree of cancer progression [197]. Furthermore, it was recently revealed that the treatment of breast cancer cells with silibinin results in the induction of apoptosis and the attenuation of both miR-21 and miR-155 levels [61] (Figure 2 and Table 3). Silibinin and its derivative (e.g., glyco-conjugated silibinin) have antioxidant activities and can protect gastric and hepatocellular carcinoma cells from xanthine oxidase-induced oxidative stress [62,63]. Therefore, it is still further required to investigate the effects of silibinin on different cancer cell types.

### 2.4. MiRNAs and Terpenoid Compounds

#### 2.4.1. Brucein D

**Figure 3:**
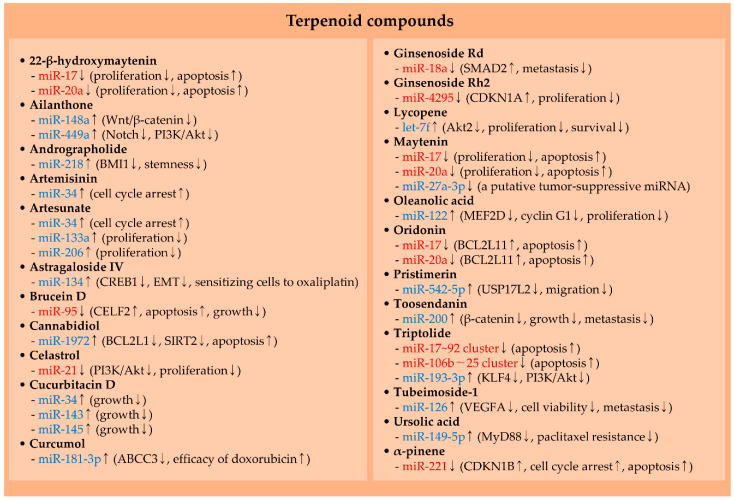
Effects of terpenoid compounds on the expression level of oncogenic miRNAs (red) and tumor-suppressive miRNAs (blue). Arrows indicate the upregulation (↑) and downregulation (↓) of miRNA levels and consequential effects on cancer. The role of miRNAs in cancer therapy with phytochemicals is described in Section 2 and Section 3.

CUGBP Elav-like family member 2 (CELF2, also called CUGBP2) is a tumor suppressor that can inhibit cancer growth and induce apoptosis. For instance, the knockdown of CELF2 leads to a decrease in apoptosis induced by ionizing radiation [198]. Also, CELF2 activates PTEN and suppresses the translation of oncogenes, such as vascular endothelial growth factor (VEGF) and cyclooxygenase-2 (COX-2) [199,200]. The efficacy of brucein D against cancer has been tested. For example, it was found that brucein D can inhibit pancreatic cancer growth by inducing ROS-mediated cell death [201]. Moreover, in hepatocellular carcinoma, it was noticed that the anti-cancer activity of brucein D is exerted by suppressing a CELF2-targeting miRNA, miR-95 [70] (Figure 3 and Table 3).

#### 2.4.2. Celastrol

Celastrol has an efficient anti-cancer property against several cancer types. In ovarian cancer, apoptosis induced by celastrol is mediated by ROS generation [202]. Additionally, celastrol hinders gastric cancer growth in vivo by modulating a number of cell-cycle and apoptosis-related genes such as CDKN1B [203]. Further evidence demonstrated that celastrol shows an anti-proliferation effect by inactivating the PI3K/Akt pathway through the downregulation of miR-21 in colorectal cancer [72] (Figure 3 and Table 3).

#### 2.4.3. Ginsenosides

Ginsenosides (G), such as G-Rd and G-Rh2, have been shown to possess anti-cancer properties through stimulating apoptosis, facilitating cell differentiation, and repressing cancer stemness, as well as angiogenesis [204,205,206]. Moreover, the association between ginsenosides and oncogenic miRNAs has been reported. In breast cancer, G-Rd shows an anti-metastatic efficacy via attenuating the levels of miR-18a, a member of the miR-17–92 cluster [75]. Their study also found that miR-18a can target SMAD family member 2 (SMAD2) [75]. It has been reported that reduced levels of SMAD2 expedite the growth and metastasis of breast cancer [207,208].

MiR-4295 was identified to accelerate cell proliferation and metastasis [209]. Also, this miRNA regulates PI3K/Akt signaling to confer resistance to cisplatin in gastric cancer cells [210]. In prostate cancer, it was demonstrated that G-Rh2 negatively regulates cell proliferation since G-Rh2 can suppress miR-4295, which directly interacts with the 3′ untranslated region (3′ UTR) of CDKN1A [76] (Figure 3 and Table 3).

#### 2.4.4. Maytenin, 22-β-Hydroxymaytenin, and Oridonin

The expression of the miR-17–92 cluster is known to be downregulated by several phytochemicals. Recently, it was noticed that the treatment of head and neck cancer cells with either maytenin or 22-β-hydroxymaytenin shows cytotoxic effects, together with a profound decrease in miR-17 and miR-20a levels. Additionally, maytenin limits metastasis of cancer in vivo, with minimal toxicity to the kidneys [78]. Moreover, oridonin diminishes the expression of miR-17 and miR-20a and triggers cell death in both doxorubicin-sensitive and -resistant leukemia cells via derepressing BCL2L11, a target mRNA of miR-17 and miR-20a [80] (Figure 3 and Table 3).

In the case of maytenin, it also reduces the levels of miR-27a-3p [78] (Figure 3 and Table 3). However, this miRNA was recently identified to inhibit EMT via negatively regulating Yes1-associated transcriptional regulator (YAP1) in head and neck cancer cells [211]. These findings suggest that maytenin adversely affects the expression of a tumor-suppressive miRNA.

#### 2.4.5. Triptolide

Similarly, in hepatocellular carcinoma cells, triptolide upregulates several tumor suppressors, including BCL2L11, PTEN, and CDKN1A, due to its capability to block the activation of ERCC excision repair 3, TFIIH core complex helicase subunit (ERCC3). Triptolide-mediated inactivation of ERCC3 represses the transcription of c-Myc and the miR-17–92 cluster [83]. In addition, the miR-106b–25 cluster levels are repressed by triptolide in a c-Myc-dependent manner [83], indicating that triptolide potentially inhibits miRNA clusters that play an oncogenic role (Figure 3 and Table 3).

#### 2.4.6. α-Pinene

In hepatocellular carcinoma, miR-221 has been well known to promote cell survival and growth via the regulation of various tumor suppressors, such as PTEN and CDKN1B [212,213]. Moreover, the downregulation of miR-221 by α-pinene was shown to participate in the suppression of hepatocellular carcinoma progression via inducing cell cycle arrest and apoptosis through activating cellular factors, including CDKN1B [86] (Figure 3 and Table 3).

## 3. Tumor-Suppressive MiRNAs Induced by Phytochemicals Currently Tested in Preclinical Studies and Clinical Trials

### 3.1. MiRNAs and Nitrogen-Containing Compounds

#### 3.1.1. 5-Aminolevulinic acid

5-Aminolevulinic acid (5-ALA) can be utilized as a sensitizer for photodynamic and sonodynamic therapies. The generation of ROS contributes to the anti-cancer effects of these 5-ALA-based therapies [214,215]. Moreover, the expression of miRNAs can be changed by 5-ALA-based therapies. 5-ALA-based photodynamic therapy increases the levels of miR-143 in cervical cancer, leading to enhanced apoptosis, along with an efficient decline of BCL-2 levels [142]. In melanoma, miR-34 is transcriptionally activated via TP53 following 5-ALA-based sonodynamic therapy [132] (Figure 1 and Table 4). This study also pointed out that miR-34 can promote TP53 activity by targeting sirtuin 1, a suppressor of TP53.

#### 3.1.2. Coptisine

One of the significantly downregulated miRNAs in hepatocellular carcinoma is miR-122. By targeting ADAM metallopeptidase domain 7 (ADAM7) and pyruvate kinase M1/2 (PKM), miR-122 negatively affects in vitro invasion, migration, and survival of hepatocellular carcinoma cells [216,217]. Additionally, the recovery of miR-122 levels efficiently blocks angiogenesis and metastasis in vivo [216]. These results suggest that miR-122 can be a beneficial candidate for miRNA-replacement therapy to manage hepatocellular carcinoma. It was also discovered that the administration of coptisine strikingly reduces cancer growth, together with an enhanced level of miR-122 in a mouse xenograft model of hepatocellular carcinoma [18] (Figure 1 and Table 4).

#### 3.1.3. Indole-3-Carbinol

Tumor-suppressive miR-34 can be transcribed in a TP53-dependent manner, thereby mediating the function of TP53. By regulating numerous target genes, such as BCL-2, miR-34 substantially blocks cell proliferation and induces apoptotic cell death, as well as cellular senescence [218]. Indole-3-carbinol has been proven to possess anti-cancer properties by regulating cell proliferation, apoptosis, and angiogenesis [219]. In addition, an experimental observation shows that indole-3-carbinol induces cell cycle arrest and TP53-dependent upregulation of miR-34 in breast cancer cells harboring wild-type TP53 [20] (Figure 1 and Table 4).

#### 3.1.4. Matrine

The detection of differentially expressed miRNAs identified that miR-22 is comparably less abundant in colorectal cancer tissues [220]. This miRNA plays a role in decelerating in vitro cell proliferation, migration, and in vivo cancer growth by targeting multiple oncogenes, such as hypoxia-inducible factor 1α (HIF-1α) and ELAV-like RNA binding protein 1 (ELAVL1) [220,221]. Matrine can facilitate cell cycle arrest and prohibit cell survival by enhancing miR-22 levels in colorectal cancer (Figure 1 and Table 4). Indeed, it was also noticed that miR-22 targets Erb-B2 receptor tyrosine kinase 3 (ERBB3) and MDS1 and EVI1 complex locus (MECOM) in colorectal cancer [43]. However, given that miR-22 can target various transcripts with different functions, it is unsurprising that miR-22 acts as either an oncogene or tumor suppressor in a cellular context-dependent manner [222].

#### 3.1.5. Sanguinarine

Another miRNA transcriptionally activated by TP53 is miR-16 that has adverse effects on cell survival due to the direct regulation of BCL-2 [223]. Additionally, transcription factor AP-4 (TFAP4), which is involved in the c-Myc-mediated EMT process, was identified as a target of miR-16 [224]. Moreover, miR-16 deters migration and invasion by inhibiting insulin-like growth factor 1 receptor (IGF1R) expression in hepatocellular carcinoma [225]. Besides, the anti-cancer effects of sanguinarine, such as cell cycle arrest and apoptosis, are caused by TP53-induced miR-16 in hepatocellular carcinoma [131] (Figure 1 and Table 4).

### 3.2. MiRNAs, Organosulfur, and Phytosterol Compounds

#### 3.2.1. Allicin

The role of miR-383-5p has been investigated in cancer. For example, it has been shown that miR-383-5p interferes with oncogenic factors, such as cellular the inhibitor of PP2A (CIP2A), which is known to stabilize c-Myc and sustain cancer progression [226,227,228]. Furthermore, recent evidence revealed that miR-383-5p is upregulated in allicin-treated gastric cancer cells, leading to the suppression of ERBB4-related oncogenic signaling such as PI3K/Akt [29] (Figure 1 and Table 4).

#### 3.2.2. Sulforaphane

In addition to the regulation of oncogenic miRNAs (Section 2.2), it was also shown that sulforaphane demethylates the promoter of miR-9 and restores the expression of miR-9 in lung cancer [129]. This study showed that sulforaphane inactivates DNA methyltransferases and attenuates the levels of histone deacetylases (HDACs) [129] (Figure 1 and Table 4). Since both sulforaphane and miR-9 are recognized to sensitize cancer cells to radiotherapy [229,230], it is feasible that miRNAs modulated by sulforaphane may contribute to enhance the effects of radiotherapy on cancer.

#### 3.2.3. β-Sitosterol-*d*-Glucoside

In breast cancer, miR-10a was determined to interrupt PI3K/Akt activation via targeting phosphatidylinositol-4,5-bisphosphate 3-kinase catalytic subunit alpha (PIK3CA), hence acting as a tumor-suppressive miRNA to suppress breast cancer progression [231]. In line with this, new findings indicated that β-sitosterol-*d*-glucoside upregulates miR-10a levels and displays anti-cancer activity through inactivating PI3K/Akt signaling [31] (Figure 1 and Table 4). Treatment with β-sitosterol-*d*-glucoside significantly diminishes cancer growth in mouse xenograft models of breast cancer [31].

### 3.3. MiRNAs and Phenolic Compounds

#### 3.3.1. Apigenin, Astragalin, and Baicalein

Multiple miRNAs acting as tumor-suppressive factors are regulated by phytochemicals in hepatocellular carcinoma. Apigenin has been proven to sensitize hepatocellular carcinoma through downregulating the levels of autophagy-related 7 (ATG7) and nuclear factor erythroid 2-related factor 2 (NFE2L2, also known as NRF2) [33,152]. Autophagy generally interrupts apoptosis induction and supports the property of cancer stem cells, thereby limiting the effects of anti-cancer drugs [4]. In addition, NFE2L2 can confer doxorubicin resistance in cancer cells via upregulating drug efflux proteins, such as P-glycoprotein (P-gp) [232]. ATG7 and NFE2L2 are downregulated by apigenin and directly targeted by miR-520b and miR-101, respectively, in hepatocellular carcinoma. Indeed, these miRNAs are increased in apigenin-treated cells, further sensitizing cancer cells to doxorubicin [33,152] (Figure 2 and Table 4).

The mechanism by which astragalin controls cancer growth was recently investigated in hepatocellular carcinoma. Astragalin increases the levels of miR-125b and impedes cancer growth in vivo (Figure 2 and Table 4). Further evidence showed that miR-125b targets hexokinase 2 (HK2) [34], which supports cell survival and the tumorigenesis of hepatocellular carcinoma [233].

In addition, treatments with baicalein show anti-cancer activity and modulate the expression of various miRNAs in hepatocellular carcinoma. One of the upregulated miRNAs by baicalein is miR-3127-5p, and the overexpression of this miRNA leads to the inhibition of cell proliferation, along with the inactivation of PI3K/Akt signaling [35] (Figure 2 and Table 4). In another study, it was demonstrated that miR-3127-5p negatively regulates Ras/ERK signaling through targeting ABL proto-oncogene 1 (ABL1) [234], supporting that miR-3127-5p acts as a tumor-suppressive miRNA.

#### 3.3.2. Brazilein

Brazilein has been supposed to exert an anti-cancer activity in cancer. Brazilein induces cell cycle arrest [37], represses invasion and migration [235], and exhibits synergistic anti-cancer effects with cisplatin in colorectal cancer cells [236]. Additionally, miR-133a is upregulated in vestibular schwannoma cells treated with brazilein (Figure 2 and Table 4). In their study, it was indicated that the activation of caspases by brazilein is abrogated in miR-133a-silencing cells, demonstrating the apoptosis-promoting potential of miR-133a [138].

#### 3.3.3. Chrysin, Curcumin, and Genistein

Curcumin and genistein exert their anti-cancer effects through modulating miR-34 levels in breast cancer and head and neck cancer, respectively [45,133] (Figure 2 and Table 4). EMT is negatively controlled by miR-34 that targets EMT-triggering factors, such as zinc finger E-box binding homeobox 1 (ZEB1) and snail family transcriptional repressor 1 (SNAI1) in breast cancer [237]. Curcumin elevates miR-34 levels, and EMT-related genes repressed by curcumin is restored by miR-34 knockdown, indicating that miR-34 mediates the effects of curcumin on EMT [133].

In addition, genistein attenuates the stemness and EMT traits of head and neck cancer. In particular, miR-34 induced by genistein directly targets RNA 2′,3′-cyclic phosphate and 5′-OH ligase (RTCB), contributing to the suppression of stemness features, including self-renewal potential [45]. This study shows that the overexpression of RTCB weakens the genistein-mediated suppression of cancer stemness (Figure 2 and Table 4).

Besides, both chrysin and curcumin can stimulate the expression of miR-132 in breast cancer cells (Figure 2 and Table 4). Additionally, the combinatorial treatment of chrysin with curcumin using nanoparticles boosts miR-132 expression and shows synergistic anti-cancer effects [38]. Through targeting forkhead box A1 (FOXA1), miR-132 shows an anti-proliferation effect on breast cancer cells [238]. Further, miR-132 negatively regulates cell proliferation and the metastasis of breast cancer via targeting Jupiter microtubule associated homolog 1 (JPT1, also called HN1). In that study, it was shown that JPT1-depleted cancer cells poorly metastasize to the lungs [239].

#### 3.3.4. Delphinidin, Epigallocatechin Gallate, and Gossypol

Delphinidin was addressed to impede invasion and migration of colorectal cancer cells without affecting proliferation status [40]. Further analyses indicated that delphinidin increases miR-204-3p expression and diminishes the levels of integrins and the activation of focal adhesion kinase (FAK) signaling (Figure 2 and Table 4). The knockdown of miR-204-3p reverses the effects of delphinidin on cell invasion, integrin levels, and FAK activity. These results indicate that miR-204-3p plays a critical role in regulating signaling pathways following delphinidin treatments [40].

Epigallocatechin gallate (EGCG) has been demonstrated to suppress the migration and stemness of cancer cells by altering miRNA expression. In nasopharyngeal carcinoma, EGCG upregulates the levels of miR-296-3p that is involved in the inactivation of STAT3, resulting in the impairment of migratory property of anoikis-resistant cells [41]. Moreover, EGCG can upregulate miR-485, which targets CD44 in cisplatin-resistant lung cancer cells, thus weakening lung cancer stemness. The in vivo administration of EGCG also shows the reduction of cancer growth and the levels of stemness factors, such as SRY-box transcription factor 2 (SOX2), Octamer-binding protein 4 (OCT4), and Nanog homeobox (NANOG) [151] (Figure 2 and Table 4).

Gossypol directly interacts with and inhibits anti-apoptotic factors, BCL-2 and BCL-2-like protein 1 (BCL2L1, also named BCL-XL), enacting apoptotic cell death in cancer [46,240]. Besides, it was uncovered that gossypol induces apoptosis and upregulates miR-15a, a BCL-2-targeting miRNA, in pituitary cancer cells [130], suggesting that gossypol can also indirectly limit BCL-2 activity (Figure 2 and Table 4).

#### 3.3.5. Hydroxygenkwanin, Isorhapontigenin, and Kaempferol

Forkhead Box M1 (FOXM1) is an EMT-promoting factor in cancer, and its knockdown hampers the invasion and migration of cancer cells [241,242]. A tumor-suppressive miRNA, miR-320a, has been known to inhibit hepatocellular carcinoma progression via targeting c-Myc and β-catenin [243,244]. Further evidence suggested that miR-320a induced by hydroxygenkwanin directly inhibits FOXM1 expression and mediates the EMT-suppressive effects of hydroxygenkwanin [47] (Figure 2 and Table 4).

Isorhapontigenin can elicit anti-cancer effects via increasing miR-137 and miR-145 [51,140,144]. It was shown that miR-137 targets SP1 and glycogen synthase kinase 3 beta (GSK3β) in bladder cancer and urothelial cancer, respectively. Thus, miR-137 suppresses anchorage-independent growth of bladder cancer and invasion of urothelial cancer [51,140]. Furthermore, isorhapontigenin escalates miR-145 levels and suppresses the sphere formation and anchorage-independent growth of patient-derived glioblastoma [144]. An additional analysis showed that miR-145 directly targets SOX2, which is involved in cyclin D1 transcription [144] (Figure 2 and Table 4).

Kaempferol was known to cause cell cycle arrest, autophagic cell death, and the inactivation of oncogenic signaling such as PI3K/Akt in cancer [245,246]. A further study on the molecular mechanism of kaempferol showed that miR-340 can be induced by kaempferol and that miR-340 knockdown abolishes the inactivation of PI3K/Akt in kaempferol-treated lung cancer cells [52] (Figure 2 and Table 4). Based on the clue that miR-340 targets sirtuin 7 (SIRT7) [247], an activator of Akt [248], it can be assumed that kaempferol negatively affects PI3K/Akt partly via the miR-340/SIRT7 axis.

#### 3.3.6. Licochalcone A and Luteolin

Licochalcone A has shown to effectively inhibit cell migration as well as EMT and activate caspases [53]. In lung cancer, licochalcone A increases miR-144-3p expression and causes apoptosis induction [143] (Figure 2 and Table 4). In this study, it was also demonstrated that miR-144-3p targets nuclear factor erythroid 2-related factor (NFE2L2, also referred to as NRF2) [143], which can activate cytoprotective autophagy to inhibit apoptotic cell death in lung cancer [249]. In line with this, miR-144-3p has been realized to inhibit SRC proto-oncogene (SRC), thereby blocking TGF-β-induced invasion [250]. By targeting c-Met, miR-144-3p also induces apoptosis to inhibit cell survival [251].

It has been investigated that luteolin also increases tumor-suppressive miRNAs to carry out its anti-cancer activity. For example, miR-34 is related to the mechanism of action of luteolin (Figure 2 and Table 4). In lung cancer, miR-34 induced by luteolin can mediate the anti-proliferative and pro-apoptotic effects of luteolin on lung cancer cells by directly controlling the expression of mouse double minute 4 (MDM4), a repressor of TP53 [54].

Pleiotrophin (PTN) can interact with neuropilin-1 receptors, stimulating downstream signaling pathways, such as PI3K and focal adhesion kinase (FAK), to facilitate cancer growth and metastasis [252]. It was suggested that miR-384 augmented by luteolin targets PTN in colorectal cancer cells [150], suggesting that luteolin suppresses colorectal cancer metastasis, in part, via the miR-384/PTN axis. Moreover, in hepatocellular carcinoma, miR-6809-5p was noticed to be elevated by luteolin, contributing to the suppression of cancer growth [154]. This study showed that miR-6809-5p represses cancer growth via targeting flotillin-1 [154], which can activate oncogenic factors such as NF-κB [253] (Figure 2 and Table 4).

#### 3.3.7. Physcion 8-*O*-β-Glucopyranoside and Quercetin

RalA-binding protein 1 (RALBP1, also called RLIP76) is a multifaceted drug transporter that mediates therapeutic resistance. Also, RALBP1 can facilitate cancer growth, angiogenesis, invasion, and metastasis [254,255,256,257]. A recent investigation showed that physcion 8-*O*-β-glucopyranoside (PG) raises the levels of miR-124, which has been validated as a tumor-suppressive miRNA in several cancer types [136] (Figure 2 and Table 4). RALBP1 can be targeted by miR-124 [136], implying a possibility that PG may sensitize cancer cells to anti-cancer agents whose efflux is catalyzed by RALBP1.

Quercetin is capable of affecting several signaling pathways that contribute to cell cycle progression, angiogenesis, and cell survival. For example, quercetin can inhibit the expression of heterogeneous nuclear ribonucleoprotein A1 (hnRNPA1), which controls mRNA exports of anti-apoptotic genes, thereby enhancing other anti-cancer agents, such as enzalutamide and JQ1 [258,259]. Moreover, recent studies pointed out that quercetin suppresses the stemness of pancreatic cancer [58,148]. The treatment of pancreatic cancer cells with quercetin leads to an increase in let-7c and NUMB endocytic adaptor protein (NUMB) levels [58] (Figure 2 and Table 4). In this study, it was proposed that the transcription of NUMB, a Notch inhibitory factor, is activated by let-7c, subsequently inhibiting the expression of Notch and stemness factors in pancreatic cancer cells. Since it was confirmed that NUMB is transcriptionally and post-transcriptionally inhibited by high mobility group AT-hook 1 (HMGA1) [260] and that Wnt/β-catenin signaling, which can increase HMGA1 levels, is repressed by let-7c [261,262], quercetin may affect Notch and stemness factor expression via the let-7c/Wnt/β-catenin/HMGA1/NUMB axis. Likewise, miR-200 induced by quercetin can contribute to repress the stemness property of pancreatic cancer cells via directly targeting Notch1 [148].

#### 3.3.8. Resveratrol and Trans-3,5,4′-trimethoxystilbene

In breast cancer, resveratrol upregulates miR-34, miR-424, and miR-503 levels in a TP53-dependent way [134] (Figure 2 and Table 4). In that study, it was shown that the downregulation of hnRNPA1 by resveratrol is directly mediated by miR-424 and miR-503. In the case of miR-34, this miRNA also indirectly represses hnRNPA1 expression [134]. Since miR-34 was reported to downregulate c-Myc, a positive regulator of hnRNPA1 expression [263,264], resveratrol may reduce hnRNPA1 levels partly via the miR-34/c-Myc axis.

Resveratrol also increases the levels of other miRNAs, thereby enhancing the chemosensitivity and inhibiting the proliferation of breast cancer cells. Resveratrol can re-sensitize resistant cells to doxorubicin by upregulating miR-122 and downregulating BCL-2 levels [135]. Since miR-122 can negatively regulate the transcription of BCL-2 via suppressing c-Myc [265], resveratrol may regulate BCL-2 levels partly via the miR-122/c-Myc axis. In addition, resveratrol can show an anti-proliferation effect by upregulating both miR-663 and miR-744, which target eukaryotic translation elongation factor 1 alpha 2 (EEF1A2) [153] (Figure 2 and Table 4).

It was also addressed that resveratrol exerts apoptosis induction and EMT suppression by upregulating miR-139-5p and miR-200, respectively (Figure 2 and Table 4). In osteosarcoma, resveratrol induces apoptosis via upregulating miR-139-5p, which targets Notch1 [141]. Resveratrol also upregulates miR-200, thereby blocking EMT progression and inducing apoptosis in colorectal cancer cells [149].

A recent study demonstrated that trans-3,5,4′-trimethoxystilbene (TMS), which is a derivative of resveratrol, sensitizes lung cancer cells to gefitinib (a selective EGFR tyrosine kinase inhibitor). The expression of miR-345 and miR-498 is significantly downregulated in gefitinib-resistant cells, and the treatment of TMS can upregulate the levels of these miRNAs. In addition, the overexpression of miR-345 and miR-498 suppresses c-Fos (FOS) and BCL-2, respectively. Further investigation showed that miR-345 and miR-498 target mitogen-activated protein kinase 1 (MAPK1) and phosphoinositide-3-kinase regulatory subunit 1 (PIK3R1), respectively, in lung cancer cells [65] (Figure 2 and Table 4).

#### 3.3.9. Silymarin

Silymarin attenuates the migration of lung cancer cells, together with the repression of HDAC activities and ZEB1 levels [64]. Further, it was demonstrated that silymarin restores the expression of miR-203, which is negatively regulated by ZEB1 [64] (Figure 2 and Table 4). These results suggest that miR-203 is one of the factors mediating the effects of silymarin on cancer cells. Indeed, it has been demonstrated that miR-203 shows its tumor-suppressive effects on lung cancer via enhancing let-7 maturation and targeting oncogenes such as SMAD3 [266,267].

### 3.4. MiRNAs and Terpenoid Compounds

#### 3.4.1. Ailanthone and Andrographolide

Ailanthone effectively restrains the proliferation, invasion, and migration of cancer cells through increasing miR-148a and miR-449a levels in breast cancer and acute myeloid leukemia cells, respectively [66,145] (Figure 3 and Table 4). In particular, miR-148a suppresses Wnt/β-catenin signaling in breast cancer [145]. In the case of miR-449a, this miRNA is involved in the restriction of Notch and PI3K/Akt signaling pathways in ailanthone-treated acute myeloid leukemia cells [66].

B lymphoma Mo-MLV insertion region 1 homolog (BMI1) advances the EMT and stemness of cancer cells via promoting NF-κB-mediated NANOG expression [268]. Recently, it was presented that andrographolide suppresses oral cancer stemness and sensitizes cancer cells to radiotherapy. The mechanism underlying the anti-cancer activity of andrographolide involves the upregulation of miR-218 that directly regulates BMI1 [67] (Figure 3 and Table 4).

#### 3.4.2. Artemisinin and Artesunate

As stated in Section 3.1.3, indole-3-carbinol upregulates miR-34 via wild-type TP53. In that study, both artemisinin and artesunate were also shown to increase miR-34 expression (Figure 3 and Table 4). The difference between indole-3-carbinol and artemisinin/artesunate is that miR-34 is increased by artemisinin and artesunate in a TP53-independent manner [20]. While further investigations are needed, it is plausible that artemisinin and artesunate may regulate miR-34 by inhibiting c-Myc, a repressor of miR-34 [68,218]. In the case of artesunate, this compound also positively regulates both miR-133a and miR-206 through ROS production in rhabdomyosarcoma cells [137] (Figure 3 and Table 4). Both miRNAs have been recognized as tumor suppressors and retard the proliferation of rhabdomyosarcoma cells [269,270].

#### 3.4.3. Astragaloside IV and Cannabidiol

Astragaloside IV subdues cancer progression via regulating multiple factors. Astragaloside IV suppresses the levels of VEGF and MMPs. PI3K/Akt signaling can also be blocked by astragaloside IV [271]. Moreover, it was demonstrated that astragaloside IV blocks the phenotype conversion of macrophages into M2-like tumor-associated macrophages (TAMs) by suppressing AMP-activated protein kinase (AMPK) activation in macrophages, thus inhibiting cancer progression [69]. In addition, astragaloside IV can upregulate the levels of miR-134, which targets CAMP-responsive element-binding protein 1 (CREB1), consequently suppressing EMT and sensitizing cancer cells to oxaliplatin [139] (Figure 3 and Table 4).

Cannabidiol activates apoptosis in neuroblastoma cells. The levels of numerous miRNAs are altered by cannabidiol treatments, and one of the upregulated miRNAs is miR-1972 (Figure 3 and Table 4). Further investigation into the relationship between apoptosis and miR-1972 showed that anti-apoptotic factors, BCL2L1 and SIRT2, are targeted by miR-1972, contributing to apoptosis induction [71].

#### 3.4.4. Cucurbitacin D, Curcumol, and Lycopene

Cucurbitacin D effectively shows anti-cancer activity toward cervical cancer. Both cell cycle arrest and apoptosis are induced by cucurbitacin D. Additionally, the expression of STAT3, c-Myc, and MMP9 are inhibited by cucurbitacin D. In addition, cucurbitacin D decelerates the growth of cervical cancer *in vivo*. Moreover, the levels of tumor-suppressive miRNAs, such as miR-34, miR-143, and miR-145, are enhanced by cucurbitacin D, suggesting that these miRNAs act as signaling mediators in cucurbitacin D-treated cells [73] (Figure 3 and Table 4).

ATP-binding cassette subfamily C member 3 (ABCC3) is one of the members of ABC transporter subfamily and is able to confer doxorubicin resistance in cancer cells by limiting intracellular concentrations of doxorubicin [74]. It was ascertained that miR-181-3p is upregulated by curcumol and that this miRNA targets ABCC3, eventually improving the efficacy of doxorubicin in breast cancer [74] (Figure 3 and Table 4). Since several anti-cancer agents are substrates for ABCC3, screening of other therapeutic agents that can be sensitized by curcumol will be beneficial to establish the new therapeutic strategy.

Similarly, lycopene impedes the proliferation and survival of prostate cancer cells [77]. It was further shown that let-7f is transcriptionally activated by lycopene and targets Akt2 [77], suggesting that the status of let-7f and Akt2 can determine the efficiency of lycopene (Figure 3 and Table 4).

#### 3.4.5. Oleanolic Acid and Pristimerin

Myocyte enhancer factor 2D (MEF2D) is a transcription factor that activates cell proliferation and migration in various cell types. For example, the knockdown of MEF2D leads to impaired proliferation and the migration of lung cancer cells [272]. Furthermore, it was noticed that the treatment of lung cancer cells with oleanolic acid upregulates miR-122 and downregulates two target mRNAs of this miRNA, namely MEF2D and cyclin G1, resulting in the suppression of cell proliferation [79] (Figure 3 and Table 4).

In breast cancer, pristimerin was observed to repress cell viability, cell cycle progression, and migration. Additional evidence demonstrated that pristimerin increases miR-542-5p levels and that this miRNA directly targets ubiquitin-specific-processing protease 17-like protein 2 (USP17L2, also referred to as DUB3) [81], which promotes migration, invasion, and metastasis of breast cancer by stabilizing SNAI1 [273] (Figure 3 and Table 4).

#### 3.4.6. Toosendanin and Triptolide

Toosendanin impedes cell proliferation, migration, invasion, and TGF-β-induced EMT. In vivo administration of toosendanin leads to the reduction of cancer growth and liver metastasis in an orthotopic implantation model of gastric cancer [82]. The molecular mechanisms of toosendanin involve miR-200, which is upregulated by toosendanin and directly targets β-catenin [82] (Figure 3 and Table 4).

In the case of triptolide, it was demonstrated that PI3K/Akt signaling is inhibited by triptolide. An increment of miR-193-3p expression by triptolide causes the downregulation of kruppel-like factor 4 (KLF4) [147], which acts as an upstream activator of PI3K/Akt signaling [274] (Figure 3 and Table 4).

#### 3.4.7. Tubeimoside-1 and Ursolic Acid

It was recently denoted that miR-126 mediates the anti-cancer effects of tubeimoside-1 by directly controlling the expression of vascular endothelial growth factor A (VEGFA) in lung cancer cells [84]. Through negatively regulating VEGFA, tubeimoside-1 inactivates ERK signaling, consequently hindering cell viability and metastasis of lung cancer cells [84] (Figure 3 and Table 4).

Myeloid differentiation primary response 88 (MyD88) is responsible for cell survival, DNA repair, and maintenance of stemness, thereby contributing to therapeutic resistance in cancer [275,276]. It was disclosed that ursolic acid reverses paclitaxel resistance by downregulating MyD88 levels in lung and breast cancer [85,146]. These studies also confirmed that miR-149-5p is upregulated in ursolic acid-treated cells and negatively affects MyD88 levels (Figure 3 and Table 4).

## 4. MiRNAs Affecting the Anti-Cancer Activity of Phytochemicals Currently Tested in Preclinical Studies and Clinical Trials

### 4.1. 1′S-1′-Acetoxychavicol Acetate

**Figure 4:**
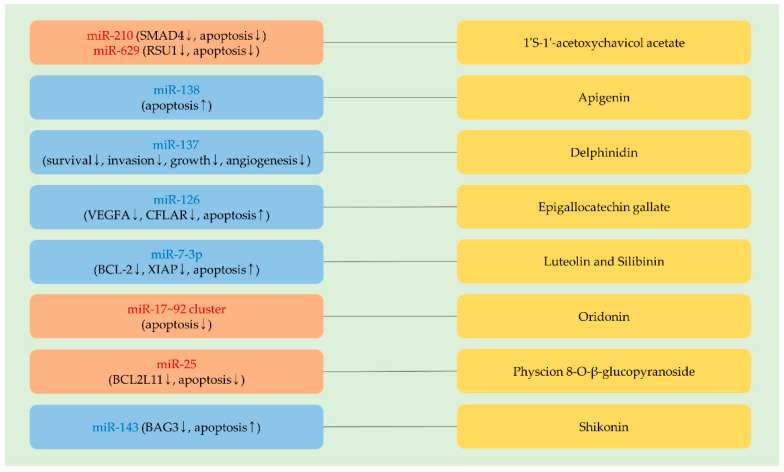
MiRNAs regulating the effectiveness of phytochemicals in cancer. MiRNAs can either sensitize (blue) or desensitize (red) cancer cells to various phytochemicals. It is described in Section 4.

While cell survival is repressed by 1′S-1′-acetoxychavicol acetate (ACA), the induction of cytoprotective autophagy can be triggered by ACA in cancer cells [32]. The inhibition of autophagy augments the efficacy of ACA [32], suggesting that autophagy-regulating miRNAs can influence the effectiveness of ACA. In cervical cancer, it was noticed that the knockdown of either miR-210 or miR-629 sensitizes cells to ACA by upregulating SMAD family member 4 (SMAD4) or Ras suppressor protein 1 (RSU1), respectively. Additional findings showed that the pro-apoptotic effects of ACA are potentiated by the overexpression of either SMAD4 or RSU1 [277,278] (Figure 4 and Table 5).

### 4.2. Apigenin

As stated in Section 3.3.1, apigenin upregulates tumor-suppressive miRNAs and enhances the efficacy of doxorubicin. Besides, it was proven that a miRNA regulates the efficacy of apigenin. The knockdown of TERT leads to an increment of miR-138 levels in neuroblastoma cells. Furthermore, the overexpression of miR-138 augments apoptosis following apigenin treatments [283] (Figure 4 and Table 5). In addition, the anti-cancer effects of apigenin are more enhanced in miR-138-overexpressing cells than in TERT-silencing cells, implying that miR-138 can downregulate multiple oncogenic factors, which block the efficacy of apigenin [283].

### 4.3. Delphinidin

The expression of miR-137 is epigenetically silenced in glioblastoma, and the overexpression of miR-137 blocks the invasion of cancer cells, indicating that miR-137 is a tumor-suppressive miRNA in glioblastoma [282]. Apoptosis induced by delphinidin is effectively intensified by miR-137 overexpression. Further investigation showed that the combination of miR-137 and delphinidin remarkably suppresses various cellular factors involved in cellular survival, invasion, growth, and angiogenesis [282] (Figure 4 and Table 5).

### 4.4. Epigallocatechin Gallate

It was illustrated that the introduction of miR-126 sensitizes lung cancer cells to doxorubicin [284]. As mentioned in Section 3.4.7, miR-126 targets VEGFA. Moreover, miR-126 can target CFLAR, reversing TRAIL resistance in cervical cancer [285]. In addition, miR-126 augments epigallocatechin gallate-induced apoptosis in osteosarcoma cells [281], indicating a potential role of miR-126 in alleviating therapeutic resistance, especially in solid cancer (Figure 4 and Table 5). However, miR-126 acts as an oncogenic miRNA in acute myeloid leukemia. By maintaining the quiescence of leukemia stem cells, miR-126 can contribute to the emergence of therapeutic resistance to daunorubicin [286].

### 4.5. Luteolin and Silibinin

The efficacy of miR-7-3p was estimated in combination with luteolin or silibinin in glioblastoma. It was shown that the anti-growth effects of both luteolin and silibinin are significantly increased by the co-administration of miR-7-3p in vivo [279] (Figure 4 and Table 5). While it remains to be elucidated how miR-7-3p enhances the sensitivity of cancer cells to luteolin and silibinin, miR-7-3p is recognized to effectively reduce the level of anti-apoptotic factors, such as BCL-2 and X-linked inhibitor of apoptosis (XIAP) [279].

### 4.6. Oridonin

Oridonin shows its cytotoxic effects via downregulating miR-17 and miR-20a (Section 2.4.4). Moreover, it was noticed that the knockdown of miR-17 or miR-20a increases apoptosis following oridonin treatments, even at a low concentration [80] (Figure 4 and Table 5). These results suggest the possibility that miRNA-based therapy is attractive to improve the therapeutic efficacy of phytochemicals.

### 4.7. Physcion 8-O-β-Glucopyranoside

In ovarian cancer, physcion 8-*O*-β-glucopyranoside (PG) adequately exhibits its anti-cancer activities by inhibiting the growth, invasion, and migration of cancer cells. However, the overexpression of miR-25 was found to attenuate the effects of PG on ovarian cancer cells [280] (Figure 4 and Table 5). In line with this, miR-25, one of the miR-106b–25 cluster members, was reported to promote ovarian cancer proliferation and downregulates a pro-apoptotic factor, BCL2L11 [287].

### 4.8. Shikonin

Several studies presented that shikonin exerts its anti-cancer effects by inducing cell cycle arrest, mitochondrial dysfunction, apoptosis, and necroptosis [288,289,290]. While shikonin can effectively constrain the growth of lung cancer in vivo, cytoprotective autophagy is also induced by shikonin treatments [289]. Indeed, the pharmacological inhibition of autophagy augments the efficacy of shikonin [289]. It was also reported that miR-143, a tumor-suppressive miRNA, is downregulated in glioblastoma stem cells treated with shikonin [60]. It was further remarked that the ectopic introduction of miR-143 directly targets BCL2-binding athanogene 3 (BAG3), an anti-apoptotic factor, and enhances the cytotoxicity of shikonin [60] (Figure 4 and Table 5). These data suggest that it is required to overcome acquired resistance to shikonin for strengthening the therapeutic efficacy of shikonin.

## 5. MiRNAs Regulating the Sensitivity of Cancer Cells to Phytochemicals Currently Used in Cancer Therapy

### 5.1. Etoposide

#### 5.1.1. MiRNAs Regulating Apoptosis and Autophagy

**Figure 5:**
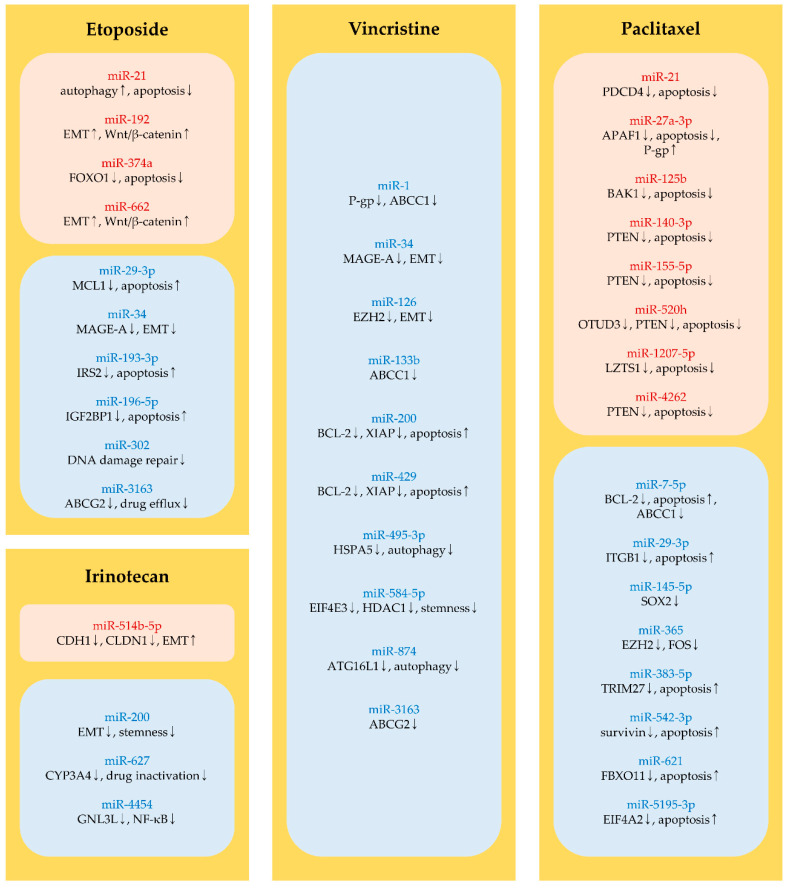
MiRNAs regulating the efficacy of plant-derived anti-cancer agents in cancer. Resistance-promoting and -suppressing miRNAs are indicated in red and blue colors, respectively. It is described in Section 5.

Recent studies demonstrated that miRNAs control the efficacy of etoposide by modulating cellular factors involved in apoptosis and cytoprotective autophagy. Etoposide resistance is advanced by miR-21 and miR-374a [291,292]. Cytoprotective autophagy is activated by miR-21; therefore, etoposide-induced apoptosis is abated by miR-21 in colorectal cancer cells [291]. In glioblastoma, miR-374a suppresses FOXO1 expression, thus blocking apoptosis induced by etoposide. The knockdown of miR-374a sensitizes cancer cells to etoposide by promoting the expression of FOXO1 [292] (Figure 5 and Table 6).

By contrast, tumor-suppressive miRNAs, such as miR-29-3p, miR-193-3p, and miR-196-5p, have been reported to target anti-apoptotic mRNAs and enhance the cytotoxicity of etoposide, showing the following: miR-29-3p sensitizes cervical cancer cells to etoposide by targeting myeloid cell leukemia sequence 1 (MCL1), a member of the BCL-2 family [293]; miR-193-3p targets insulin receptor substrate 2 (IRS2) and improves the effectiveness of etoposide in osteosarcoma cells [294]; miR-196-5p escalates etoposide-induced apoptosis via inhibiting insulin-like growth factor 2 mRNA-binding protein 1 (IGF2BP1) [295] (Figure 5 and Table 6).

#### 5.1.2. MiRNAs Regulating EMT and Wnt/β-Catenin Signaling

The efficacy of etoposide can be regulated by miRNAs that control EMT and Wnt/β-catenin signaling. In lung cancer cells, both miR-192 and miR-662 potentiate cell invasion and anchorage-independent growth. Additionally, the expression of genes that activate EMT and Wnt/β-catenin signaling is enhanced by these miRNAs, hence contributing to the appearance of etoposide resistance [301] (Figure 5 and Table 6).

It was also demonstrated that melanoma-associated antigen-A (MAGE-A) promotes EMT in retinoblastoma cells. In that study, miR-34 was validated to target MAGE-A and negatively regulate EMT-related factors, thereby bringing about an improvement of etoposide cytotoxicity [309] (Figure 5 and Table 6).

#### 5.1.3. MiRNAs Regulating DNA Damage Repair

It was recognized that miR-302 directly targets DNA repair protein RAD52 homolog (RAD52) in leukemia cells [315]. Since one of the causes of therapeutic resistance is DNA damage repair pathways, the overexpression of miR-302 can sensitize cells to etoposide via repressing RAD52, which generally limits the efficacy of cancer therapies [315,326] (Figure 5 and Table 6).

#### 5.1.4. A miRNA Regulating a Drug Transporter Level

ABCG2 (also referred to as breast cancer resistance protein (BCRP)) is responsible for therapeutic resistance by mediating the efflux of several anti-cancer agents, including etoposide [327]. It was recently reported that miR-3163 decelerates the proliferation of retinoblastoma cancer stem cells (RCSC), indicating that this miRNA has a tumor-suppressive property. Also, the anti-proliferative effects of etoposide are enhanced in miR-3163-overexpressing RCSC. Indeed, this miRNA was confirmed to target ABCG2 [323] (Figure 5 and Table 6).

### 5.2. Irinotecan

#### 5.2.1. MiR-200

As described in Section 3.3 and Section 3.4, miR-200 suppresses cell viability, EMT, and stemness, suggesting that this miRNA can be used as a resistance-suppressive miRNA. In fact, it was shown that the combined treatment of irinotecan and miR-200 forcefully restrict the growth of colorectal cancer with a minimized systemic toxicity in mouse xenograft models [313] (Figure 5 and Table 6).

#### 5.2.2. MiR-514b-5p

The metastasis of colorectal cancer is facilitated by miR-514b-5p. Functional experiments showed that miR-514b-5p triggers EMT and invasion of cancer cells via targeting cadherin 1 (CDH1, also named E-cadherin 1) and claudin 1 (CLDN1). Further, it was shown that the lentiviral delivery of miR-514b-5p decreases the efficacy of irinotecan in mouse xenograft models [302] (Figure 5 and Table 6).

#### 5.2.3. MiR-627

Irinotecan is metabolized into inactive forms by cytochrome P450 family 3 subfamily A member 4 (CYP3A4) [328]. A recent study demonstrated that miR-627 can directly target CYP3A4, thereby enhancing the effects of irinotecan on growth inhibition and apoptosis induction in colorectal cancer cells [15] (Figure 5 and Table 6). In this study, it was also shown that calcitriol, a synthetic vitamin D, reduces CYP3A4 levels via upregulating miR-627 and enhances the anti-cancer efficacy of irinotecan.

#### 5.2.4. MiR-4454

Moreover, it was recently found that miR-4454 expression is repressed in irinotecan-resistant colorectal cancer cells [324]. Further analyses indicated that the restoration of miR-4454 effectively sensitizes cells to irinotecan, hence obstructing cell growth, migration, and invasion of irinotecan-resistant cells (Figure 5 and Table 6). A mechanism underlying miR-4454-mediated sensitization of cancer cells involves the downregulation of guanine nucleotide-binding protein-like 3-like protein (GNL3L), an activator of NF-κB transcription activity [324].

### 5.3. Paclitaxel

#### 5.3.1. MiRNAs Negatively Regulating Apoptosis

Multiple apoptosis-inhibiting miRNAs weaken paclitaxel activity toward cancer cells. In breast cancer, miR-21 and miR-125b target programmed cell death 4 (PDCD4) and BCL2-antagonist/killer 1 (BAK1), respectively, eventually aggravating paclitaxel resistance [296,299]. In addition, leucine zipper tumor suppressor 1 (LZTS1), a repressor of PI3K/Akt signaling [329], is known to be targeted by miR-1207-5p. Therefore, miR-1207-5p is capable of diminishing paclitaxel cytotoxicity toward breast cancer cells [304] (Figure 5 and Table 6).

Moreover, miR-27a-3p is increased by the hypoxic condition and advances resistance to paclitaxel by targeting apoptotic protease-activating Factor 1 (APAF1), a mediator of cytochrome c-dependent caspase activations, in ovarian cancer cells [297] (Figure 5 and Table 6).

The sensitivity of cancer cells to paclitaxel can be abolished by PTEN inhibition. In chordoma, both miR-140-3p and miR-155-5p contribute to exacerbating paclitaxel resistance via directly targeting PTEN [300]. It was also noted that miR-4262 expression is intensively augmented in paclitaxel-resistant lung cancer cells compared to paclitaxel-sensitive parental cells. By directly blocking PTEN, miR-4262 reduces the efficacy of paclitaxel. The co-treatment with paclitaxel and miR-4262 inhibitors synergistically inhibits the growth of cancer, along with the inactivation of Akt and GSK3β in xenograft models in which paclitaxel-resistant cells were subcutaneously injected [305] (Figure 5 and Table 6).

OTU domain-containing protein 3 (OTUD3) is recognized as a deubiquitinase of PTEN, thus acting as a tumor suppressor by stabilizing PTEN. By targeting OTUD3, miR-520h can contribute to paclitaxel resistance via indirectly suppressing PTEN. As a matter of fact, the silencing of miR-520h re-sensitizes resistant cells to paclitaxel by augmenting apoptosis induction [303] (Figure 5 and Table 6).

#### 5.3.2. MiRNAs Positively Regulating Apoptosis

In breast cancer, apoptosis following paclitaxel treatments is boosted by miR-7-5p, miR-542-3p, miR-621, and miR-5195-3p via directly modulating BCL-2, survivin, F-box protein 11 (FBXO11), and eukaryotic translation initiation factor 4A2 (EIF4A2), respectively [307,321,325]. The paclitaxel-sensitizing effects of miR-7-5p are reversed by the restoration of BCL-2 in breast cancer cells [307]. By targeting survivin, miR-542-3p potentiates anti-cancer effects of paclitaxel in vitro and in vivo [319]. In addition, miR-621 increases TP53 activity by repressing FBXO11. Paclitaxel-induced apoptosis in miR-621-overexpressing cells is attenuated by the upregulation of FBXO11 [321]. In the case of miR-5195-3p, the expression of this miRNA is downregulated in paclitaxel-resistant cell lines and tissues derived from breast cancer patients. The overexpression of EIF4A2 abrogates apoptosis induction in paclitaxel-resistant cells [325] (Figure 5 and Table 6).

It has been demonstrated that integrin-mediated signaling impairs paclitaxel-induced apoptosis via inhibiting cytochrome c release [330]. A recent study uncovered that integrin β1 (ITGB1) and miR-29-3p levels are increased and decreased, respectively, in paclitaxel-resistant nasopharyngeal cancer cells. The overexpression of miR-29-3p re-sensitizes resistant cells to paclitaxel by repressing ITGB1. The knockdown of miR-29-3p was found to facilitate the growth of paclitaxel-resistant cells in vivo, indicating that miR-29-3p plays a critical role in regulating the effectiveness of paclitaxel [308] (Figure 5 and Table 6).

Tripartite motif-containing protein 27 (TRIM27) inhibits PTEN, hence activating PI3K/Akt signaling. The knockdown of TRIM27 is known to induce apoptosis in cancer cells [331]. TRIM27 is overexpressed in ovarian cancer tissues and correlated with the unfavorable prognosis of patients with ovarian cancer [317]. It was also noticed that miR-383-5p is downregulated in ovarian cancer tissues and is able to target TRIM27. The overexpression of miR-383-5p enhances paclitaxel efficacy via increasing apoptosis induction [317] (Figure 5 and Table 6).

#### 5.3.3. MiRNAs Inhibiting EMT- and Stemness-Related Factors

Notch signaling is known to induce and maintain the stemness of cancer cells. Enhancer of zeste homolog 2 (EZH2) activates Notch signaling, thereby expanding cancer stem cell populations [332]. In addition, proto-oncogene c-Fos (FOS) promotes EMT and the expression of several stemness factors, such as Notch1 and SOX2 [333]. Both EZH2 and FOS were validated as target mRNAs of miR-365, and the upregulation of miR-365 inhibits cell invasion, migration, and survival. Additionally, the sensitivity of cells to paclitaxel is enhanced by miR-365 in endometrial cancer [316] (Figure 5 and Table 6).

SOX2, a stemness factor, can promote EMT through activating STAT3 signaling and maintain self-renewal potential [334]. SOX2 is directly regulated by miR-145-5p in breast cancer [312]. Both miR-145-5p overexpression and SOX2 knockdown reverse paclitaxel resistance. The efficacy of paclitaxel in miR-145-5p-overexpressing cells is abrogated by the restoration of SOX2 levels [312] (Figure 5 and Table 6).

#### 5.3.4. MiRNAs Regulating the Level of Drug Transporters

In addition to BCL-2 (Section 5.3.2), miR-7-5p was also investigated to target ABCC1 (also called multidrug resistance protein 1, MRP1) [307]. These results demonstrated that miR-7-5p can sensitize cancer cells to paclitaxel via augmenting both pro-apoptotic pathways and intracellular paclitaxel levels (Figure 5 and Table 6).

As mentioned above, miR-27a-3p acts as an anti-apoptotic factor (Section 5.3.1). In addition to this role, miR-27a-3p was found to be upregulated in paclitaxel-resistant ovarian cancer cells. By targeting homeodomain-interacting protein kinase-2 (HIPK2), miR-27a-3p can indirectly stimulate P-gp levels, leading to paclitaxel resistance. The knockdown of miR-27a-3p makes paclitaxel-resistant cells more susceptible to paclitaxel [298] (Figure 5 and Table 6).

### 5.4. Vincristine

#### 5.4.1. MiRNAs Regulating Apoptosis and Autophagy

The expression of miR-200 and miR-429 is downregulated in vincristine-resistant gastric cancer cells. These miRNAs are able to augment the induction of apoptosis following the treatment of resistant cells with vincristine. It was further pointed out that BCL-2 and XIAP are targeted by both miRNAs, suggesting that the efficiency of vincristine can be impeded by the alteration of miR-200 and miR-429 levels [314] (Figure 5 and Table 6).

The anti-cancer activity of vincristine can be augmented by autophagy-inhibiting miRNAs. In gastric cancer, miR-495-3p inhibits cytoprotective autophagy via targeting heat shock protein 70 family protein 5 (HSPA5, also called GRP78) [318]. The inhibition of HSPA5 by miR-495-3p suppresses autophagy via triggering mTOR signaling, improving the cytotoxicity of vincristine. Moreover, the lentiviral delivery of miR-495-3p efficiently decreases the growth of vincristine-resistant cells in vivo [318]. In addition, miR-874 inhibits autophagy via targeting ATG16L1, a positive regulator of autophagosome formation [322]. Both miR-874 overexpression and ATG16L1 knockdown elevate the efficacy of vincristine in gastric cancer cells [322] (Figure 5 and Table 6).

#### 5.4.2. MiRNAs Inhibiting EMT- and Stemness-Related Factors

As stated in Section 5.1.2, the miR-34/MAGE-A axis affects etoposide cytotoxicity. In that study, it was also confirmed that the sensitivity of cells to vincristine is significantly enhanced and diminished in miR-34-overexpressing and -silencing retinoblastoma cells, respectively [309] (Figure 5 and Table 6).

In addition to miR-365 (see Section 5.3.3), EZH2 is also directly regulated by miR-126 in gastric cancer cells. It was remarked that the level of miR-126 is diminished in vincristine-resistant cells and that miR-126 overexpression can re-sensitize resistant cells to vincristine. The sensitization of cells to vincristine is also thoroughly observed in EZH2 silencing cells [310] (Figure 5 and Table 6).

The growth of medulloblastoma is known to be inhibited by miR-584-5p. In addition, the overexpression of miR-584-5p was identified to suppress the self-renewal of medulloblastoma cells owing to its ability to target EIF4E3 and HDAC1. Certainly, the silencing of EIF4E3 and HDAC1 also represses the self-renewal capacity of medulloblastoma. Further, the ectopic expression of miR-584-5p intensifies the anti-cancer efficacy of vincristine [320] (Figure 5 and Table 6).

#### 5.4.3. MiRNAs Regulating the Intracellular Concentration of Vincristine

Sorcin was proven to induce the expression of P-gp and ABCC1, thus contributing to therapeutic resistance [335,336]. Notably, it was demonstrated that there is a negative correlation between sorcin and miR-1 levels in vincristine-resistant gastric cancer cells [306]. The levels of P-gp and ABCC1 are markedly upregulated in vincristine-resistant cells. The overexpression of miR-1 downregulates sorcin, consequently inhibiting the levels of P-gp and ABCC1 as well. Due to this ability, miR-1 can reverse vincristine-resistant in gastric cancer [306] (Figure 5 and Table 6).

Furthermore, ABCC1 is directly targeted by miR-133b. The intracellular concentration of vincristine is increased by miR-133b in colorectal cancer cells. Therefore, the inhibitory effects of vincristine on colony formation, cell survival, and cancer growth are significantly advanced by miR-133b [311] (Figure 5 and Table 6).

By suppressing ABCG2, miR-3163 sensitizes retinoblastoma cells not only to etoposide, but also to vincristine (also see Section 5.1.4). It was also validated that miR-3163 can enhance the efficacy of other anti-cancer agents, such as cisplatin, suggesting that miR-3163 plays a significant role in sensitizing cancer cells to therapeutic agents [323] (Figure 5 and Table 6).

## 6. Conclusions

The accumulating evidence presented here indicates that phytochemicals have valuable potentials as therapeutic agents against cancer. Single treatments of phytochemicals effectively regulate cellular signaling pathways to exert their anti-cancer activities. Additionally, phytochemicals sensitize cancer cells to other conventional drugs, such as doxorubicin, indicating a great possibility of their use in combination with other anti-cancer agents to maximize therapeutic responses for managing cancer.

Various anti-cancer activities of phytochemicals are mediated by altering the expression of miRNAs in cancer cells. As expected, oncogenic and tumor-suppressive miRNAs are generally downregulated and upregulated, respectively, by phytochemicals. This modulation of miRNA expression by phytochemicals plays a critical part in regulating the levels of cellular factors and restoring drug sensitivity to other anti-cancer agents.

Current challenges for cancer therapy with phytochemicals are, for example, the low solubility of compounds, cancer-specific delivery of compounds, and limited penetration of compounds into cancer. To overcome these impediments, the delivery of phytochemicals using nanoparticles has been investigated [38,337]. Moreover, the co-delivery of phytochemicals using nanoparticles displays synergistic effects on cancer cells (Section 3.3.3), demonstrating an opportunity to develop a new combination strategy for cancer therapy.

There is an unexpected modulation of miRNA levels (Section 2.1.6 and Section 2.4.4). Since there are over 2000 human miRNAs [338], it is feasible that a substantial number of miRNAs can be errantly modulated following cancer therapy, so alternative oncogenic factors can be activated, ultimately contributing to the acquired resistance to phytochemicals. Since drug resistance is another challenge in cancer therapy, comprehensive analyses of miRNA-mediated signaling pathways following the application of phytochemical compounds will enable the identification of the molecular targets to develop therapeutic strategies that prevent and overcome drug resistance. As mentioned in Section 4 and Section 5, the alteration of miRNA expression definitely regulates the sensitivity of cancer cells to phytochemicals and derivatives. From this perspective, a combination of miRNA modulators (mimics or inhibitors) and phytochemicals will also offer a new strategy for cancer therapy.

While miRNAs play critical roles in cancer, it has been realized that some miRNAs behave differently depending on the type of cancer. Examples of the dual role of miRNAs are discussed in this review (see Section 2.1.1 and Section 2.3.6). Additionally, as mentioned above, the treatment of cancer with therapeutic compounds can change miRNA levels in an unanticipated way. Therefore, it would be more promising if specific miRNAs are selectively targeted. Butylcycloheptyl prodiginine is a derivative of prodiginine, a natural compound from Gammaproteobacteria, and inhibits miR-21 levels by binding to miR-21 precursors and blocking Dicer-mediated maturation [339]. Additionally, we mentioned the ability of sophocarpine to target miR-21 precursors in Section 2.1.8. Unlike mature miRNAs, the sequence of miRNA precursors is typically not conserved among various miRNAs. Therefore, targeting miRNA precursors makes it possible to discriminate among miRNAs [340]. It suggests that phytochemicals (as well as natural compounds from other resources) can be developed as specific miRNA target agents to design a novel strategy for cancer therapy. Further investigations will be necessary to screen the effects of natural compounds on the Dicer-mediated maturation of other miRNAs. Advanced knowledge through further studies that uncover the role of phytochemicals and miRNAs will help to establish a clinically valuable therapeutic strategy against cancer.

## Figures and Tables

**Table 1:** The list of phytochemicals and derivatives currently tested in preclinical studies and clinical trials.

Phytochemical	Source of Phytochemical (NCT Number, Condition/Disease, Recruitment Status if Applicable)	Ref.
Nitrogen-containing compounds (alkaloids and amino acids)		
5-Aminolevulinic acid *	A precursor of tetrapyrroles in higher plants (NCT02144077, basal cell carcinoma, active)	[16]
Berberine *	An isoquinoline alkaloid in plants, such as barberry and goldenseal (NCT03281096, colorectal adenomas, recruiting)	[17]
Coptisine	A protoberberine alkaloid in *Rhizoma coptidis*	[18]
Evodiamine	An indoloquinazoline alkaloid in *Evodia rutaecarpa*	[19]
Indole-3-carbinol	An indole alkaloid in vegetables such as cauliflower	[20]
Matrine	A quinolizidine alkaloid in the root of *Sophora flavescens*	[21]
Neferine	A bisbenzylisoquinline alkaloid in the seed embryo of *Nelumbo nucifera*	[22]
Nitidine chloride	A benzophenanthridine alkaloid in the roots of *Zanthoxylum nitidum*	[23]
Piperlongumine	An alkaloid from *Piper longum* Linn	[24]
Sanguinarine	A benzophenanthridine alkaloid in Papaveraceae plants	[25]
Sinomenine	An isoquinoline alkaloid in the dry roots and stems of *Sinomenium acutum*	[26]
Sophocarpine	A tetracyclic quinolizidine alkaloid in *Sophora alopecuroides* Linn	[27]
Swainsonine	An indolizidine alkaloid in *Swainsona canescens*	[28]
Organosulfur and phytosterol compounds		
Allicin	An organosulfur compound from garlic (Allium sativum)	[29]
Sulforaphane *	An isothiocyanate abundant in cruciferous vegetables such as broccoli sprouts (NCT03182959, head and neck cancer, active)	[30]
β-sitosterol-*d*-glucoside	A phytosterol from sweet potato	[31]
Phenolic compounds		
1′S-1′-acetoxychavicol acetate	A phenylpropanoid from *Alpinia conchigera*	[32]
Apigenin	A flavone from fruits (e.g., oranges) and vegetables (e.g., onions)	[33]
Astragalin	A flavonoid broadly present in food such as lotus leaves	[34]
Baicalein(aglycone of baicalin)	A flavonoid from *Scutellaria radix*	[35]
Baicalin	A flavonoid from the root of *Scutellaria baicalensis* Georgi	[36]
Brazilein	A polyphenolic compound from *Caesalpinia sappan*	[37]
Chrysin	A flavone from several plants, including *Oroxylum indicum*	[38]
Curcumin *	A polyphenolic curcuminoid in *Curcuma longa* (turmeric plant) (NCT02064673, prostate cancer, recruiting)	[39]
Delphinidin	A flavonoid in fruits and vegetables such as tomatoes	[40]
Epigallocatechin gallate *	A polyphenol in green tea (NCT02891538, colorectal cancer, recruiting)	[41]
Formononetin	An isoflavone in the root of *Astragalus membranaceus*	[42]
Galangin	A flavonoid in *Alpinia officinarum*	[43]
Gambogic acid	A xanthonoid from *Garcinia hanburyi* trees	[44]
Genistein *	An isoflavone and phytoestrogen primarily in Soybeans (NCT00118040, bladder cancer, active)	[45]
Gossypol (AT-101) *	A polyphenol from cotton roots and seeds (NCT01633541, advanced laryngeal cancer, active)	[46]
Hydroxygenkwanin	A flavonoid from *Daphne genkwa*	[47]
Honokiol	A polyphenol in the genus Magnolia	[48]
Icariin	A flavonoid in Epimedium (Horny Goat Weed and Yin Yang Huo)	[49]
Isoliquiritigenin	A chalcone from *Glycyrrhizae radix*	[50]
Isorhapontigenin	A stilbene from *Gnetum cleistostachyum*	[51]
Kaempferol	A flavonoid in various plants such as Brussels sprouts	[52]
Licochalcone A	A chalcone from *Glycyrrhiza uralensis*	[53]
Luteolin	A flavonoid in several vegetables such as cabbage	[54]
Physcion 8-*O*-β-glucopyranoside	An anthraquinone in *Rumex japonicus* Houtt	[55]
Procyanidin	A polyphenol in dietary fruits such as grapes	[56]
Puerarin	An isoflavone in the root of Pueraria (*Radix puerariae*)	[57]
Quercetin *	A flavonoid in fruits and vegetables, such as onions and broccoli (NCT01912820, prostate cancer, active)	[58]
Resveratrol	A stilbenoid in grapes and red wine, etc	[59]
Shikonin	A naphthoquinone from *Lithospermum erythrorhizon*	[60]
Silibinin	A polyphenolic flavonoid in milk thistle (*Silybum marianum*)	[61,62,63]
Silymarin	*A mixture of flavonolignans* from *Silybum marianum* L. Gaertn.	[64]
Trans-3,5,4′-trimethoxystilbene	A derivative of resveratrol	[65]
Terpenoid compounds		
Ailanthone	A quassinoid from *Ailanthus altissima*	[66]
Andrographolide *	A diterpene lactone from *Andrographis paniculate* (NCT04196075, advanced esophageal cancer, recruiting)	[67]
Artemisinin	A sesquiterpene lactone from *Artemisia annua*	[68]
Artesunate *	A derivative of artemisinin (NCT02633098, colorectal cancer, recruiting)	[68]
Astragaloside IV	A pentacyclic triterpenoid from *Astragali radix*	[69]
Brucein D	A triterpene quassinoid in *Brucea javanica* fruit	[70]
Cannabidiol *	A terpenophenolic compound from *Cannabis sativa* (NCT04428203, prostate cancer, recruiting)	[71]
Celastrol	A triterpene in *Tripterygium wilfordii*	[72]
Cucurbitacin D	A derivative of cucurbitacin that is a tetracyclic triterpene from the Cucurbitaceae family	[73]
Curcumol	A sesquiterpenoid from *Rhizoma Curcumae*	[74]
Ginsenoside Rd and Rh2	Triterpene saponins in *Panax* genus	[75,76]
Lycopene *	A carotenoid from fruits such as tomatoes (NCT03167268, colorectal cancer, recruiting)	[77]
Maytenin and 22-β-hydroxymaytenin	Quinone-methide triterpenes in *Maytenus ilicifolia*	[78]
Oleanolic acid	A pentacyclic triterpene in herbs and vegetables	[79]
Oridonin	A diterpenoid in genus *Isodon* plants	[80]
Pristimerin	A triterpenoid from the Celastraceae and Hippocrateaceae families	[81]
Toosendanin	A triterpenoid from *Melia toosendan* Sieb et Zucc	[82]
Triptolide *	A diterpene triepoxide in *Tripterygium wilfordii* Hook F (NCT03129139, advanced solid tumors, recruiting)	[83]
Tubeimoside-1	A triterpenoid saponin from *Bolbostemma paniculatum*	[84]
Ursolic acid *	A pentacyclic triterpene in plants such as apples (NCT04403568, prostate cancer, not yet recruiting)	[85]
α-pinene	A monoterpene in pine needles	[86]

* indicates phytochemical compounds currently in clinical trials on cancer. The national clinical trial (NCT) number, condition/disease, and recruitment status registered in ClinicalTrials.gov are referred.

**Table 2:** Anti-cancer compounds derived from plants that are currently used in clinical practice.

Anti-Cancer Agent	Source	Primary Anti-Cancer Action/Application *	Ref.
Etoposide	A derivative of podophyllotoxin, a non-alkaloid lignan that is isolated from *Podophyllum peltatum*	Topoisomerase II inhibition/Approved for small cell lung cancer and testicular cancer	[10]
Irinotecan	A derivative of camptothecin that is a monoterpene indole alkaloid from *Camptotheca acuminata*	Topoisomerase I inhibition/Approved for colorectal cancer	[9]
Paclitaxel	A terpenoid isolated from the Pacific yew tree	Stabilization of microtubule polymer/Approved for AIDS-related Kaposi sarcoma, breast cancer, non-small cell lung cancer, and ovarian cancer	[11]
Vincristine	A vinca alkaloid from *Catharanthus roseus*	An inhibition of microtubule polymerization/Approved for acute leukemia. Also used to treat Hodgkin lymphoma, neuroblastoma, non-Hodgkin lymphoma, rhabdomyosarcoma, and Wilms tumor	[10]

* information on the drug application is from the National Cancer Institute.

**Table 3:** Oncogenic miRNAs downregulated by phytochemicals in cancer.

miRNA	Phytochemical (A Type of Cancer)	Effective in Vitro Concentration of Phytochemical/Treatment Time	Effective in Vivo Dose of Phytochemical in Mouse Models of Cancers (A Route of Administration)	Ref.
miR-17–92 cluster	Ginsenoside Rd (breast cancer)	≥50 μM/72 h	50 mg/kg (intraperitoneal)	[75]
	Maytenin and 22-β-hydroxymaytenin (head and neck squamous cell carcinomas)	1.5–1.6 μM/24 h * (maytenin), 1.9–2.5 μM/24 h * (22-β-hydroxymaytenin)	2 mg/kg (maytenin, intraperitoneal)	[78]
	Nitidine chloride (chronic myeloid leukemia)	≥4 μM/72 h	-	[91]
	Oridonin (myelogenous leukemia)	≥5 μM/24 h	10–15 mg/kg (intraperitoneal)	[80]
	Procyanidin (lung cancer)	≥10 μM/24 h	56–112 mg/kg (oral)	[56]
	Resveratrol (breast cancer)	≥6.25 μM/48 h	25–100 mg/kg (intraperitoneal)	[92]
	Swainsonine (glioblastoma)	≥20 μM/12 h	-	[28]
	Triptolide (hepatocellular carcinoma)	≥50 nM/48 h	0.2 mg/kg (intraperitoneal)	[83]
miR-21	Celastrol (colorectal cancer)	3.2 μM/72 h *	-	[72]
	Curcumin (osteosarcoma)	≥2.5 μM/72 h	-	[39]
	Formononetin (bladder cancer)	≥50 μM/24 h	-	[42]
	Galangin (cholangiocarcinoma)	≥50 μM/24 h	-	[43]
	Gambogic acid (colorectal cancer)	≥1 μM/48 h	-	[44]
	Honokiol (osteosarcoma)	≥1 μM/24 h	-	[48]
	Puerarin (hepatocellular carcinoma)	≥50 μM/48 h	40 mg/kg (intravenous)	[57]
	Silibinin (breast cancer)	200 μM/48 h *	-	[61]
	Sophocarpine (head and neck cancer)	1–1.5 μM/48 h *	5 mg/kg (intravenous)	[27]
	Sulforaphane (colorectal cancer)	≥5 μM/72 h	-	[30]
	Sulforaphane (glioblastoma)	≥5 μM/24 h	-	[93]
miR-23	Baicalin (colorectal cancer)	165.5 μM/24 h *	50–100 mg/kg (intraperitoneal)	[36]
	Sinomenine (prostate cancer)	≥0.25 mM/24 h	-	[26]
	Sulforaphane (breast cancer)	≥2.5 μM/24 h	-	[94]
miR-32	Isoliquiritigenin (nasopharyngeal cancer)	≥12.5 μM/48 h	25–100 mg/kg (oral)	[50]
miR-95	Brucein D (hepatocellular carcinoma)	≥0.5 μM/72 h	1.5 mg/kg (intraperitoneal)	[70]
miR-99a–125b cluster	Berberine (multiple myeloma)	75 μM/48 h **	-	[17]
miR-103-3p	Physcion 8-*O*-β-glucopyranoside (gastric cancer)	≥10 μM/96 h	30–50 mg/kg (intraperitoneal)	[55]
miR-106b–25 cluster	Epigallocatechin gallate (breast cancer)	≥10 μM/24 h	100 mg/kg (oral)	[95]
	Matrine (gastric cancer)	≥10 μM/24 h	-	[96]
	Triptolide (hepatocellular carcinoma)	≥50 nM/48 h	0.2 mg/kg (intraperitoneal)	[83]
miR-155	Silibinin (breast cancer)	200 μM/48 h *	-	[61]
miR-183-96-182 cluster	Curcumin (breast cancer)	20 μM/24 h *	-	[97]
	Matrine (papillary thyroid cancer)	≥1 mM/48 h	-	[21]
	Sanguinarine (gastric cancer)	150–200 μM/48 h *	5–10 mg/kg (intraperitoneal)	[25]
miR-196b	Resveratrol (acute lymphoblastic leukemia)	≥25 μM/72 h	-	[98]
miR-217	Baicalin (colorectal cancer)	≥10 μM/24 h	-	[99]
miR-221	Resveratrol (melanoma)	50 μM/24 h **	30 mg/kg (intraperitoneal)	[59]
	α-pinene (hepatocellular carcinoma)	≥16 μM/24 h	-	[86]
miR-222	Resveratrol (thyroid cancer)	≥0.5 μM/72 h	-	[100]
miR-223	Genistein (pancreatic cancer)	50–60 μM/72 h *	-	[101]
miR-301-3p	Isoliquiritigenin (melanoma)	≥10 μM/24 h	20 mg/kg (intraperitoneal)	[102]
	Luteolin (pancreatic cancer)	23.3 μM/96 h *	-	[103]
miR-324-5p	Sinomenine (breast cancer)	≥0.25 mM/24 h	-	[104]
miR-374a	Isoliquiritigenin (breast cancer)	≥6.25 μM/72 h	-	[105]
	Neferine (breast cancer)	≥4 μM/24 h	-	[22]
miR-382	Sulforaphane (breast cancer)	≥2.5 μM/24 h	-	[94]
miR-429	Berberine (colorectal cancer)	≥4 μM/72 h	-	[90]
	Evodiamine (colorectal cancer)	≥3 μM/72 h	-	
miR-625-3p	Icariin (thyroid cancer)	≥20 μM/24 h	-	[49]
miR-1290	Resveratrol (acute lymphoblastic leukemia)	≥25 μM/72 h	-	[98]
miR-4295	Ginsenoside Rh2 (prostate cancer)	≥16.6 μM/96 h	-	[76]
miR-7641	Curcumin (bladder cancer)	≥10 μM/48 h	-	[106]

* the half maximal inhibitory concentration (IC50) at an indicated treatment time. ** tests only at an indicated concentration.

**Table 4:** Tumor-suppressive miRNAs modulated by phytochemicals.

miRNA	Phytochemical (A Type of Cancer)	Effective in Vitro Concentration of Phytochemical/Treatment Time	Effective in Vivo Dose of Phytochemical in Mouse Models of Cancers (A Route of Administration)	Ref.
Let-7c	Quercetin (pancreatic cancer)	50 μM/48 h ***	-	[58]
Let-7f	Lycopene (prostate cancer)	≥10 μM/48 h	-	[77]
miR-9	Sulforaphane (lung cancer)	≥2.5 μM/120 h	-	[129]
miR-10a	β-sitosterol-*d*-glucoside (breast cancer)	30.82–49.76 μM/24 h **	60 mg–120 mg/kg (intragastric)	[31]
miR-15a	Gossypol (pituitary cancer)	≥5 μM/96 h	-	[130]
miR-16	Sanguinarine (hepatocellular carcinoma)	≥0.5 μM/48 h	10 mg/kg (intraperitoneal)	[131]
miR-22	Matrine (colorectal cancer)	≥0.25 mM/72 h	-	[43]
miR-27a-3p *	Maytenin (head and neck cancer)	1.5–1.6 μM/24 h **	2 mg/kg (intraperitoneal)	[78]
miR-29-3p *	Sanguinarine (gastric cancer)	150–200 μM/48 h **	5–10 mg/kg (intraperitoneal)	[25]
miR-34	5-Aminolevulinic acid (melanoma)	1 mM/4 h + ultrasound	200 mg/kg (intraperitoneal) + ultrasound	[132]
	Artemisinin and Artesunate (breast cancer)	300 μM/48 h *** (artemisinin), ≥5 μM/48 h (artesunate)	-	[20]
	Cucurbitacin D (cervical cancer)	≥0.05 μM/72 h	1 mg/kg (intra-tumoral)	[73]
	Curcumin (breast cancer)	≥20 μM/48 h	-	[133]
	Genistein (head and neck cancer)	≥20 μM/24 h	25 mg–50 mg/kg (oral)	[45]
	Indole-3-carbinol (breast cancer)	≥200 μM/48 h	-	[20]
	Luteolin (lung cancer)	40 μM/72 h **	50 mg–200 mg/kg (intragastric)	[54]
	Resveratrol (breast cancer)	≥12.5 μM/72 h	-	[134]
miR-101	Apigenin (hepatocellular carcinoma)	10 μM/48 h ***	-	[33]
miR-122	Coptisine (hepatocellular carcinoma)	≥3.1 μM/24 h	150 mg/kg (oral)	[18]
	Oleanolic acid (lung cancer)	≥65 μM/48 h	120 mg/kg (oral)	[79]
	Resveratrol (breast cancer)	≥100 μM/24 h	-	[135]
miR-124	Physcion 8-*O*-β-glucopyranoside (melanoma)	≥10 μM/24 h	-	[136]
miR-125b	Astragalin (hepatocellular carcinoma)	≥11 μM/48 h	10–20 mg/kg (oral)	[34]
miR-126	Tubeimoside-1 (lung cancer)	≥10 μM/48 h	-	[84]
miR-132	Chrysin and Curcumin (breast cancer)	≥20 μM/48 h (chrysin), ≥10 μM/48 h (curcumin)	-	[38]
miR-133a	Artesunate (rhabdomyosarcoma)	≥5 μM/24 h	25 mg/kg (intraperitoneal)	[137]
	Brazilein (vestibular schwannoma)	≥10 μM/96 h	-	[138]
miR-134	Astragaloside IV (colorectal cancer)	≥6.4 μM/48 h	-	[139]
miR-137	Isorhapontigenin (bladder cancer)	10 μM/24 h ***	150 mg/kg (intraperitoneal)	[51]
	Isorhapontigenin (urothelial cancer)	≥5 μM/24 h	-	[140]
miR-139-5p	Resveratrol (osteosarcoma)	≥5 μM/48 h	-	[141]
miR-143	5-Aminolevulinic acid (cervical cancer)	≥0.25 μM + photodynamic therapy/24 h	-	[142]
	Cucurbitacin D (cervical cancer)	≥0.05 μM/72 h	1 mg/kg (intra-tumoral)	[73]
miR-144-3p	Licochalcone A (lung cancer)	≥10 μM/48 h	-	[143]
miR-145	Cucurbitacin D (cervical cancer)	≥0.05 μM/72 h	1 mg/kg (intra-tumoral)	[73]
	Isorhapontigenin (glioblastoma)	≥10 μM/24 h	-	[144]
miR-148a	Ailanthone (breast cancer)	≥5 μM/48 h	-	[145]
miR-149-5p	Ursolic acid (breast cancer)	20 μM/48 h ***	10 mg/kg (intraperitoneal)	[85]
	Ursolic acid (lung cancer)	≥5 μM/48 h	Treated cells with ursolic acid at 20 μM for 72 h before implanting in mice	[146]
miR-181-3p	Curcumol (breast cancer)	≥254 μM/48 h	20 mg/kg of curcumol + 2.5 mg/kg of doxorubicin (intraperitoneal)	[74]
miR-193-3p	Triptolide (nephroblastoma)	≥10 nM/72 h	-	[147]
miR-200	Quercetin (pancreatic cancer)	50 μM/72 h ***	-	[148]
	Resveratrol (colorectal cancer)	≥25 μM/48 h	-	[149]
	Toosendanin (gastric cancer)	≥0.5 μM/48 h	0.2 mg/kg (intraperitoneal)	[82]
miR-203	Silymarin (lung cancer)	≥10 μM/24 h	-	[64]
miR-204-3p	Delphinidin (colorectal cancer)	≥25 μM/24 h	Treated cells with delphinidin at 100 μM for 24 h before implanting in mice	[40]
miR-206	Artesunate (rhabdomyosarcoma)	≥5 μM/24 h	25 mg/kg (intraperitoneal)	[137]
miR-218	Andrographolide (oral cancer)	≥6.25 μM/24 h	10 mg/kg (oral)	[67]
miR-296-3p	Epigallocatechin gallate (nasopharyngeal cancer)	≥20 μM/24 h	-	[41]
miR-320a	Hydroxygenkwanin (hepatocellular carcinoma)	≥10 μM/72 h	1 mg/kg (intraperitoneal)	[47]
miR-340	Kaempferol (lung cancer)	≥20 μM	-	[52]
miR-345	Trans-3,5,4′-trimethoxystilbene (lung cancer)	≥0.5 μM/72 h	30 mg/kg (oral)	[65]
miR-383-5p	Allicin (gastric cancer)	62 μM/48 h ***	-	[29]
miR-384	Luteolin (colorectal cancer)	≥10 μM/48 h	100 mg/kg (intragastric)	[150]
miR-424	Resveratrol (breast cancer)	≥12.5 μM/72 h	-	[134]
miR-449a	Ailanthone (acute myeloid leukemia)	≥0.5 μM/48 h	-	[66]
miR-485	Epigallocatechin gallate (lung cancer)	≥10 μM/24 h	20 mg/kg (intraperitoneal)	[151]
miR-498	Trans-3,5,4′-trimethoxystilbene (lung cancer)	≥0.5 μM/72 h	30 mg/kg (oral)	[65]
miR-503	Resveratrol (breast cancer)	≥12.5 μM/72 h	-	[134]
miR-520b	Apigenin (hepatocellular carcinoma)	≥10 μM/48 h	50 mg/kg (intraperitoneal)	[152]
miR-542-5p	Pristimerin (breast cancer)	≥1 μM/24 h	-	[81]
miR-663	Resveratrol (breast cancer)	100 μM/24 h ***	-	[153]
miR-744	Resveratrol (breast cancer)	100 μM/24 h ***	-	[153]
miR-1972	Cannabidiol (neuroblastoma)	≥5 μM/24 h	-	[71]
miR-3127-5p	Baicalein (hepatocellular carcinoma)	≥20 μM/24 h	-	[35]
miR-6809-5p	Luteolin (hepatocellular carcinoma)	≥10 μM/120 h	50 mg/kg (intraperitoneal)	[154]

* indicates tumor-suppressive miRNAs that are downregulated by phytochemicals. ** the half maximal inhibitory concentration (IC50) at an indicated treatment time. *** tests only at an indicated concentration.

**Table 5:** MiRNAs controlling anti-cancer activity of phytochemicals.

miRNA	Phytochemical (A Type of Cancer)	In Vitro Finding	In Vivo Experiment Condition	Ref.
miR-7-3p	Luteolin and Silibinin (glioblastoma)	Treatment with luteolin (20 μM/24 h) or silibinin (50 μM/24 h) induces apoptosis and miR-7-3p expression	10 mg/kg (luteolin, oral) or 200 mg/kg (silibinin, oral) with and without miR-7-3p (50 μg, intravenous)	[279]
miR-25	Physcion 8-*O*-β-glucopyranoside (ovarian cancer)	Overexpression of miR-25 reduces the cytotoxicity of physcion 8-*O*-β-glucopyranoside (10 μM)	-	[280]
miR-17–92 cluster	Oridonin (myelogenous leukemia)	Inhibition of miR-17 or miR-20a enhances the cytotoxicity of oridonin (2.5 and 5 μM for 72 h)	10–15 mg/kg (oridonin, intraperitoneal)	[80]
miR-126	Epigallocatechin gallate (osteosarcoma)	Overexpression of miR-126 potentiates the cytotoxicity of epigallocatechin gallate (100 μM for 48 h)	-	[281]
miR-137	Delphinidin (glioblastoma)	Overexpression of miR-137 enhances apoptosis induced by delphinidin (50 μM for 24 h)	-	[282]
miR-138	Apigenin (neuroblastoma)	Ectopic expression of miR-138 enhances apoptosis induced by apigenin (100 μM for 24 h)	10 μg/mouse (apigenin, intra-tumoral) + hTERT shRNA plasmid or miR-138 mimic	[283]
miR-143	Shikonin (glioblastoma)	Overexpression of miR-143 enhances apoptosis induced by shikonin (2 μM for 24 h)	2 mg/kg (shikonin, intraperitoneal) in mice bearing miR-143-overexpressing cells	[60]
miR-210	1′S-1′-acetoxychavicol acetate (cervical cancer)	Knockdown of miR-210 potentiates the cytotoxicity of 1′S-1′-acetoxychavicol acetate (5–20 μM for 48 h)	-	[277]
miR-629	1′S-1′-acetoxychavicol acetate (cervical cancer)	Knockdown of miR-629 potentiates the cytotoxicity of 1′S-1′-acetoxychavicol acetate (5–20 μM for 48 h)	-	[278]

**Table 6:** MiRNAs involving in the regulation of efficacy of plant-derived anti-cancer agents.

miRNAs	Phytochemicals (A Type of Cancer)	In Vitro Finding	In Vivo Experiment Condition	Ref.
Cancer resistance-promoting miRNAs				
miR-21	Etoposide (colorectal cancer)	3.97 μM/72 h * (miR-control-overexpressing cells), 12.7 μM/72 h * (miR-21-overexpressing cells)	-	[291]
	Paclitaxel (breast cancer)	≈ 6 μM/48 h * (anti-miR-control-treated cells), ≈ 3 μM/48 h * (anti-miR-21-treated cells)	1 mg/kg (paclitaxel, intravenous) + anti-miR-21 (intra-tumoral)	[296]
miR-27a-3p	Paclitaxel (ovarian cancer)	Silencing of miR-27a-3p increases the cytotoxicity of paclitaxel (4, 8, and 12 μM for 7 days)	5 mg/kg (paclitaxel) in mice bearing miR-27a-3p-overexpressing cells	[297]
	Paclitaxel (ovarian cancer)	Silencing of miR-27a-3p increases the cytotoxicity of paclitaxel (0.04–23 μM for 48 h)	-	[298]
miR-125b	Paclitaxel (breast cancer)	Overexpression of miR-125b reduces the cytotoxicity of paclitaxel (4, 8, and 16 nM for 48 h)	-	[299]
miR-140-3p	Paclitaxel (chordoma)	Silencing of miR-140-3p or miR-155-5p increases the cytotoxicity of paclitaxel (10 μM for 24–72 h)	-	[300]
miR-155-5p				
miR-192	Etoposide (lung cancer)	Knockdown of miR-192 sensitizes cells to etoposide (≤100 μM for 24 h)	-	[301]
miR-374a	Etoposide (glioblastoma)	Knockdown of miR-374a sensitizes cells to etoposide (0.5–8 μM for 48 h)	-	[292]
miR-514b-5p	Irinotecan (colorectal cancer)	Overexpression of miR-514b-5p reduces the cytotoxicity of irinotecan (25, 50, and 100 μM for 48 h)	40 mg/kg (irinotecan) in mice bearing miR-514b-5p-overexpressing cells	[302]
miR-520h	Paclitaxel (breast cancer)	Overexpression of miR-520h reduces the cytotoxicity of paclitaxel (1, 5, and 10 nM for 24 h)	-	[303]
miR-662	Etoposide (lung cancer)	Knockdown of miR-662 sensitizes cells to etoposide (≤100 μM for 24 h)	-	[301]
miR-1207-5p	Paclitaxel (breast cancer)	Knockdown of miR-1207-5p sensitizes cells to paclitaxel (10 nM for 5 days)	-	[304]
miR-4262	Paclitaxel (lung cancer)	Overexpression of miR-4262 reduces the cytotoxicity of paclitaxel (4 and 8 μM)	Paclitaxel + anti-miR-4262 (intravenous)	[305]
Cancer resistance-suppressing miRNAs				
miR-1	Vincristine (gastric cancer)	Knockdown of miR-1 reduces the cytotoxicity of vincristine (10 and 20 μM)	-	[306]
miR-7-5p	Paclitaxel (breast cancer)	25–35 μM/72 h * (miR-control-overexpressing cells), 5–15 μM/72 h * (miR-7-5p-overexpressing cells)	-	[307]
miR-29-3p	Etoposide (cervical cancer)	Overexpression of miR-29-3p enhances the cytotoxicity of etoposide (60 μM for 48 h)	-	[293]
	Paclitaxel (nasopharyngeal cancer)	6.4–7.5 nM/96 h * (miR-control-overexpressing cells), 0.7–0.8 nM/96 h * (miR-29-3p-overexpressing cells)	5 mg/kg (paclitaxel, intraperitoneal) in mice bearing miR-29-3p inhibitor treated cells	[308]
miR-34	Etoposide (retinoblastoma)	Overexpression of miR-34 enhances the cytotoxicity of etoposide (35 μM for 48 h)	-	[309]
	Vincristine (retinoblastoma)	Overexpression of miR-34 enhances the cytotoxicity of vincristine (130 nM for 48 h)	-	[309]
miR-126	Vincristine (gastric cancer)	Overexpression of miR-126 enhances the cytotoxicity of vincristine (1.2, 6, and 12 μM for 48 h)	-	[310]
miR-133b	Vincristine (colorectal cancer)	420 μM/24 h * (miR-control-overexpressing cells), 120 μM/24 h * (miR-133b-overexpressing cells)	-	[311]
miR-145-5p	Paclitaxel (breast cancer)	≈ 6 μM/24 h * (miR-control-overexpressing cells), ≤3 μM/24 h * (miR-145-5p-overexpressing cells)	miR-145-5p mimic (intra-tumoral) in mice bearing paclitaxel-resistant cells	[312]
miR-193-3p	Etoposide (osteosarcoma)	Overexpression of miR-193-3p enhances the cytotoxicity of etoposide at IC50/72 h	-	[294]
miR-196-5p	Etoposide (hepatocellular carcinoma)	Overexpression of miR-196-5p increases apoptosis induced by etoposide (50 μM for 16 h)	-	[295]
miR-200	Irinotecan (colorectal cancer)	Delivery of miR-200 using nanoparticles increases apoptosis after treatment with irinotecan (incorporated in liposomes) for 48 h	100 mg/kg (irinotecan, intravenous) + 1.25 mg/kg (miR-200, intravenous)	[313]
	Vincristine (gastric cancer)	Overexpression of miR-200 increases apoptosis induced by vincristine (4 μM for 48 h)	-	[314]
miR-302	Etoposide (leukemia)	Overexpression of miR-302 enhances the cytotoxicity of etoposide (20, 50, 100, and 200 μM)	20 mg/kg (etoposide, intraperitoneal) in mice bearing miR-302-overexpressing cells	[315]
miR-365	Paclitaxel (endometrial cancer)	Overexpression of miR-365 enhances the cytotoxicity of paclitaxel (200 and 300 nM for 24 h)	-	[316]
miR-383-5p	Paclitaxel (ovarian cancer)	Overexpression of miR-383-5p enhances the cytotoxicity of paclitaxel (≤5 μM)	Subcutaneous injection of miR-383-5p-overexpressing cells	[317]
miR-429	Vincristine (gastric cancer)	Overexpression of miR-429 increases apoptosis induced by vincristine (4 μM for 48 h)	-	[314]
miR-495-3p	Vincristine (gastric cancer)	Overexpression of miR-495-3p enhances the cytotoxicity of vincristine (≤1.65 μM for 24 h)	Subcutaneous injection of miR-495-3p-overexpressing cells	[318]
miR-542-3p	Paclitaxel (breast cancer)	Overexpression of miR-542-3p increases apoptosis induced by paclitaxel (3 nM for 24 h)	6 mg/kg (paclitaxel, intraperitoneal) + 5μg (miR-542-3p, intra-tumoral)	[319]
miR-584-5p	Vincristine (medulloblastoma)	Overexpression of miR-584-5p enhances the cytotoxicity of vincristine (≤1 μM for 72 h)	0.5 mg/kg (vincristine, intraperitoneal) in mice bearing miR-584-5p-overexpressing cells	[320]
miR-621	Paclitaxel (breast cancer)	Overexpression of miR-621 promotes apoptosis induced by paclitaxel (20 μM for 24 h)	15 mg/kg (paclitaxel, intraperitoneal) in mice bearing miR-621-overexpressing cells	[321]
miR-627	Irinotecan (colorectal cancer)	Overexpression of miR-627 enhances the cytotoxicity of irinotecan (5, 10, and 20 μM for 48 h)	50 mg/kg (irinotecan, intraperitoneal) + 0.4 μg (calcitriol, intraperitoneal)	[15]
miR-874	Vincristine (gastric cancer)	≈ 4 μM/48 h * (anti-miR-control treated cells), ≈ 2 μM /48 h * (anti-miR-874 treated cells)	-	[322]
miR-3163	Etoposide (retinoblastoma)	0.57 μM/72 h * (miR-control-overexpressing cells), 0.39 μM/72 h * (miR-3163-overexpressing cells)	-	[323]
	Vincristine (retinoblastoma)	1.27 μM/72 h * (miR-control-overexpressing cells), 0.77 μM/72 h * (miR-3163-overexpressing cells)	-	[323]
miR-4454	Irinotecan (colorectal cancer)	Knockdown of miR-4454 reduces apoptosis induced by irinotecan (10 μM for 48 h)	Subcutaneous injection of miR-4454 expressing cells	[324]
miR-5195-3p	Paclitaxel (breast cancer)	Overexpression of miR-5195-3p enhances the cytotoxicity of paclitaxel (2.5–10 μM)	-	[325]

* the half maximal inhibitory concentration (IC50) at indicated treatment time.
